# The cell-permeant antioxidant D-thiol ester D-cysteine ethyl ester overcomes physical dependence to morphine in male Sprague Dawley rats

**DOI:** 10.3389/fphar.2024.1444574

**Published:** 2024-08-26

**Authors:** Paulina M. Getsy, Gregory A. Coffee, James N. Bates, Theodore Parran, Lee Hoffer, Santhosh M. Baby, Peter M. MacFarlane, Zackery T. Knauss, Derek S. Damron, Yee-Hsee Hsieh, Jason A. Bubier, Devin Mueller, Stephen J. Lewis

**Affiliations:** ^1^ Department of Pediatrics, Case Western Reserve University, Cleveland, OH, United States; ^2^ Department of Anesthesiology, University of Iowa Hospitals and Clinics, Iowa City, IA, United States; ^3^ Center for Medical Education, Case Western Reserve University School of Medicine, Cleveland, OH, United States; ^4^ Department of Anthropology, Case Western Reserve University, Cleveland, OH, United States; ^5^ Section of Biology, Galleon Pharmaceuticals, Inc., Horsham, PA, United States; ^6^ Department of Biological Sciences, Kent State University, Kent, OH, United States; ^7^ Division of Pulmonary, Critical Care and Sleep Medicine, Case Western Reserve University, Cleveland, OH, United States; ^8^ The Jackson Laboratory, Bar Harbor, ME, United States; ^9^ Department of Pharmacology, Case Western Reserve University, Cleveland, OH, United States; ^10^ Functional Electrical Stimulation Center, Case Western Reserve University, Cleveland, OH, United States

**Keywords:** opioids, morphine, naloxone, physical dependence, withdrawal, D-cysteine, D-cysteine ethyl ester, d-serine

## Abstract

The ability of morphine to decrease cysteine transport into neurons by inhibition of excitatory amino acid transporter 3 (EAA3) may be a key molecular mechanism underlying the acquisition of physical and psychological dependence to morphine. This study examined whether co-administration of the cell-penetrant antioxidant D-thiol ester, D-cysteine ethyl ester (D-CYSee), with morphine, would diminish the development of physical dependence to morphine in male Sprague Dawley rats. Systemic administration of the opioid receptor antagonist, naloxone (NLX), elicited pronounced withdrawal signs (e.g., wet-dog shakes, jumps, rears, circling) in rats that received a subcutaneous depot of morphine (150 mg/kg, SC) for 36 h and continuous intravenous infusion of vehicle (20 μL/h, IV). The NLX-precipitated withdrawal signs were reduced in rats that received an infusion of D-CYSee, but not D-cysteine, (both at 20.8 μmol/kg/h, IV) for the full 36 h. NLX elicited pronounced withdrawal signs in rats treated for 48 h with morphine (150 mg/kg, SC), plus continuous infusion of vehicle (20 μL/h, IV) that began at the 36 h timepoint of morphine treatment. The NLX-precipitated withdrawal signs were reduced in rats that received a 12 h infusion of D-CYSee, but not D-cysteine, (both at 20.8 μmol/kg/h, IV) that began at the 36 h timepoint of morphine treatment. These findings suggest that D-CYSee may attenuate the development of physical dependence to morphine and reverse established dependence to the opioid in male Sprague Dawley rats. Alternatively, D-CYSee may simply suppress the processes responsible for NLX-precipitated withdrawal. Nonetheless, D-CYSee and analogues may be novel therapeutics for the treatment of opioid use disorders.

## Introduction

Clinicians face serious challenges when treating pain in subjects with opioid use disorder (OUD) and substance use disorders (SUDs) (McCarberg, 2011; Heberlein et al., 2012; Garcia-Portilla et al., 2014; Hah et al., 2017; Dydyk et al., 2022; Horn et al., 2022; Matson et al., 2022; Dydyk et al., 2023). To minimize potential misuse of pain medications, clinicians need to figure out how to provide opioid-induced analgesia without producing euphoria, psychological-physical dependence, and hyperalgesia (Benyamin et al., 2008; Morgan and Christie, 2011; Volkow et al., 2018; Mercadante et al., 2019; Manhapra, 2022; Preux et al., 2022; Balanaser et al., 2023). With respect to moderate to severe OUD, terminology from the *American Psychiatric Associatio*n for opioid addiction in 10%–20% of people with SUD liability (Diagnostic and Statistical Manual of Mental Disorders, 2013), key problems are (i) how to treat opioid withdrawal, as current strategies have major weaknesses, (ii) how to effectively manage subjects who have gone through withdrawal and require therapeutics to block dependence and euphoria, with available drugs (e.g., methadone, buprenorphine) having strengths, but also major weaknesses, and (iii) how to minimize opioid-induced euphoria and dependence in those with moderate to severe OUD who are currently off opioids but need opioid analgesia (Humphreys et al., 2022; Jiménez-Fernández et al., 2022; Torres-Lockhart et al., 2022; Alvarez-Perez et al., 2023; Biancuzzi et al., 2023; Frankeberger et al., 2023). In treating SUD due to opioids, cannabinoids, alcohol, psycho-stimulants or benzodiazepines, a vital problem is how to provide an effective therapeutic that will attenuate dopamine-mediated euphoria of each brain-reward drug, in addition to treating addictive disease states (Stephan and Parsa, 2016; Horsfall and Sprague, 2017; Bechara et al., 2019; Serafini and Zachariou, 2019; Chartoff and Connery, 2024). In regard to making opioid analgesics safer, there are no drugs that improve analgesic efficacy of opioids. Ideally, drugs should (i) promote opioid analgesia while preventing hyperalgesia, (ii) prevent acquisition of psychological/physical dependence to opioids, and (iii) prevent/overcome opioid-induced respiratory depression (OIRD) (Dydyk et al., 2022; Horn et al., 2022; Matson et al., 2022; Dydyk et al., 2023).

The development of morphine-induced dependence may involve redox-based changes in global DNA methylation and retrotransposon transcription *via* μ-opioid receptor (μ-OR)-mediated inhibition of excitatory amino acid transporter type 3 (EAA3)-facilitated cysteine uptake into central neurons (Trivedi et al., 2014). Previous research suggested that co-administration of cell-permeant analogues of L-cysteine, such as L-cysteine ethyl ester (L-CYSee) (Goto et al., 1983; Hisadome et al., 1986a; Hisadome et al., 1986b, Hisadome et al., 1988; Hisadome et al., 1990; Servin et al., 1988; Schöneich et al., 1992; Hobbs et al., 1993; Fukui et al., 1994; Galanakis et al., 2004; Perissinotti et al., 2005; Ding and Demple, 1998; Mosier-Boss and Lieberman, 2005; Defonsi Lestard et al., 2013; Mendoza et al., 2013; Arias et al., 2019), may diminish acquisition of dependence to morphine and reverse established dependence. Indeed, we reported that intravenous infusion of L-CYSee, but not L-cysteine or L-serine ethyl ester, prevented acquisition of morphine dependence in rats and reversed acquired dependence to morphine (Bates et al., 2023). The inability of L-cysteine or L-serine ethyl ester (oxygen atom rather than sulfur atom as in L-CYSee) suggests that the efficacy of L-CYSee is due to cell penetrability into brain regions vital to expression of morphine dependence, and points to the vital role of thiol pathways in the efficacy of the L-thiol ester (Bates et al., 2023). These results are complimented by our findings that L-CYSee (Lewis et al., 2022), L-cysteine methyl ester (Getsy et al., 2022a), other thiol esters and related compounds, such as S-nitrosothiols (Gaston et al., 2021; Jenkins et al., 2021; Getsy et al., 2022b; Getsy et al., 2022c; Getsy et al., 2022d; Getsy et al., 2022e; Getsy et al., 2022f), and the free radical scavenger, Tempol (Baby et al., 2021a; Baby et al., 2021b), prevent and/or reverse the adverse actions of morphine and fentanyl on breathing, arterial blood-gas chemistry (pH, pCO_2_, pO_2_, sO_2_) and Alveolar-arterial gradient (index of alveolar gas-exchange) in rats without affecting opioid-induced analgesia or sedation.

The chronic ingestion or co-ingestion of drugs with abuse liability, such as opioids (morphine, heroin, fentanyl and remifentanil), cocaine, alcohol, cannabinoids and methamphetamine, causes oxidative/nitrosative stress and adverse changes in redox and glutathione homeostasis by decreased activities of superoxide dismutase, catalase, and glutathione peroxidase (Sharma et al., 2007; Neri et al., 2015; Go et al., 2017; Womersley et al., 2019; Berríos-Cárcamo et al., 2020; Soltaninejad et al., 2024). With respect to opioids, these adverse redox changes have been implicated in the etiologies of the key aspects of opioid use disorder (OUD), including physical dependence, addiction, tolerance, and the development of hyperalgesia (Cunha-Oliveira, 2008; Salvemini, 2009; Salvemini and Neumann, 2010; Uys et al., 2014; Zahmatkesh et al., 2017; Guleken et al., 2020; Su et al., 2021; Vorspan et al., 2021; Newman et al., 2022; Viola et al., 2023, see Supplementary Table S1 for reference list). Therefore, therapeutics that effectively overcome the redox imbalance in individuals with OUD would be of clinical benefit. While we do not know the mechanisms by which L- and D-CYSee exert their effects against OIRD, it is tempting to assume that the ability of these cell-penetrant thiol esters and parent thiols to act as intracellular reducing-antioxidant agents plays a key role. Endogenous D-cysteine (Seckler and Lewis, 2020; Homma et al., 2022; Roychaudhuri et al., 2022; Roychaudhuri, 2023; Souza et al., 2023) and L-cysteine (Go and Jones, 2011; Paulsen and Carroll, 2013; Bak and Weerapana, 2015; Paul et al., 2018; Held, 2020) exert multi-factorial effects due to their ability to affect intracellular redox status. L-CYSee (Lewis et al., 2022) and D-CYSee (Getsy et al., 2022d; Getsy et al., 2022e) effectively reduce and reverse OIRD in rats, and D-CYSee overcomes physical dependence to fentanyl in rats (Bates et al., 2024a) and conditioned place preference (active drug seeking) to fentanyl in rats (Knauss et al., 2023).

Stereoisomeric configuration is often a critical factor in allowing drugs to bind to and affect the activities of functional proteins (Waldeck, 1993; Cudic and Otvos, 2002; Brocks and Mehvar, 2003; Kasprzyk-Hordern, 2010). Our above-mentioned publications with L-,D-thiol esters show that the L-isomers have pharmacological activity not shared by the D-isomers in overcoming OIRD, most likely because of stereoisomeric configuration and disparate abilities to enter metabolic/enzymatic pathways (Getsy et al., 2022a; Getsy et al., 2022d; Getsy et al., 2022e; Lewis et al., 2022). We are at early stages of determining if L-D-thiol esters have different activities against OUD. As mentioned, L-CYSee prevented development of physical dependence to morphine in male rats and overcame established dependence in these rats (Bates et al., 2023). Accordingly, the objective of the present study was to determine whether D-CYSee is also able to overcome physical dependence to morphine. Our findings that D-CYSee is as effective as L-CYSee raises important mechanistic questions and furthers the argument that cell-penetrant antioxidants may be a novel class of compounds to treat OUD. Furthermore, on-going studies are showing that L-CYSee and D-CYSee act synergistically such that substantially lower doses of each produce full effects by mechanisms under investigation.

## Methods

### Permissions, rats and surgical procedures

All studies were done according to the NIH Guide for Care and Use of Laboratory Animals (NIH Pub No. 80–23) revised in 1996, and in compliance with ARRIVE (Animal Research: Reporting of *In Vivo* Experiments) guidelines (http://www.nc3rs.org.uk/page. asp? id=1357). All protocols involving rats were approved by the Animal Care and Use Committees of Galleon Pharmaceuticals and Case Western Reserve University. A total of 432 adult male Sprague Dawley rats purchased from *Harlan Industries* (Madison, WI) were used (Supplementary Table S2). Body weights of rats in each study group are described below (there were no between group differences in body weights in any study group). Rats were given 5 days to recover from transportation before surgery. (+)-Morphine sulfate was purchased from *Baxter Healthcare* (Deerfield, IL). Powders of D-CYSee HCl, D-cysteine HCl, and D-serine HCl were from *Sigma-Aldrich* (St. Louis, MO). D-SERee HCl was from *Neta Scientific* (Hainesport, NJ). Powders were divided into 100 mg amounts under N_2_ gas and stored at 4°C. Solutions of these compounds (dissolved in normal saline and brought to pH 7.2 with 0.1 M NaOH) were prepared immediately before use. Naloxone HCl (NLX; *Sigma-Aldrich*, St. Louis, MO) was dissolved in normal saline. On the day of the experiment, arterial and venous catheters were flushed with 0.3 mL of phosphate-buffered saline (0.1 M, pH 7.4) 3–4 h before commencing the study. All studies were done in a quiet room with relative humidity of 50% ± 2% and temperature of 21.3°C ± 0.2°C. Each rat was used only once.

## Protocols to determine the effects of D-CYSee on physical dependence to morphine–Prevention of morphine dependence

### Behavioral studies

At 2 p.m. on the day of surgery, groups of rats received a jugular vein catheter (PE-10 connected to PE-50) under 2%–3% isoflurane anesthesia (Henderson et al., 2013; Henderson et al., 2014; May et al., 2013a; May et al., 2013b). The jugular vein catheter was connected to a primed ALZET osmotic minipump (Model 2002, ALZA Corporation, CA) positioned at the back of the neck to infuse vehicle (20 μL/h, IV) or D-cysteine, D-CYSee, D-serine or D-SERee (all at 20.8 μmol/kg/h, IV) (Jarrott et al., 1987; Jarrott et al., 1988; Lewis et al., 1988a; Lewis et al., 1989). All wounds were sutured closed and the rats were returned to their warmed home cages. Physical dependence was induced by a slow-release subcutaneous depot of morphine emulsion (150 mg/kg, SC) injected at the left side of the neck (Lee and Fennessy, 1970; Laska and Fennessy, 1976; Laska and Fennessy, 1977; Lewis et al., 1988b). Morphine base was precipitated from a solution of (+)-morphine sulfate by titrating to pH 9 with 1 mM NaOH. After several distilled water washes, pure base was collected in a filter funnel and dried. Morphine slow-release emulsion was prepared by suspending a weighed amount of base in liquid paraffin and *Arlacel A*. This mixture was emulsified with an equal volume of normal saline (Collier et al., 1972). After 35.5 h of morphine exposure, the rats were placed in individual opaque boxes and after 30 min, they received an intraperitoneal (IP) injection of NLX (1.5 mg/kg) and behaviors were scored for 45 min by three scorers blinded to treatments. Scored phenomena were: Jumping behavior–all four paws of the ground–jumps; Wet dog shakes -whole body shakes as if to shed water from fur; Rearing behavior–rearing on hind legs–rears; Episodes of fore-paw licking–FPL; Circling–Complete 360° rotation; Writhes–full body contortion; Episodes of sneezing–abrupt expulsion of air that disturbed the fine bedding material-sneezes.

### Plethysmography ventilatory studies

Rats were prepared as above except that they also received a second catheter into the jugular vein (Getsy et al., 2022c; Getsy et al., 2022f) to inject NLX. After 35 h, the rats were placed into individual whole body plethysmography chambers (Getsy et al., 2022b; Getsy et al., 2022c; Getsy et al., 2022f) and the free end of the exteriorized venous catheter was connected to a swivel assembly in the lid of each chamber. After 60 min, the rats were injected with NLX (1.5 mg/kg), IV). Ventilatory parameters were recorded and the number of apneas >1.5 s in duration were determined by internal *FinePointe* software (DSI, Harvard Bioscience, Inc., St. Paul, MN) (Lewis et al., 2022).

## Cardiovascular studies

Rats were prepared as above except that they also received a second catheter into the jugular vein (Getsy et al., 2022c; Getsy et al., 2022f) to give NLX, and a catheter into a femoral artery to continuously record mean arterial blood pressure (MAP) and heart rate as described previously (Kanbar et al., 2010; Davisson et al., 2014; Brognara et al., 2016; Gaston et al., 2020). After 35 h, rats were placed in individual opaque plastic boxes and the jugular vein catheter was connected to an injection line to give NLX. The arterial line was connected to tubing attached to a computer-coupled pressure transducer (*Cabe Lab*, *Inc*.) to record pulsatile arterial blood pressure. After 60 min, rats were injected with NLX (1.5 mg/kg, IV) and MAP and heart rate were recorded continuously for 90 min.

## Body Temperature and Body Weight Studies

Groups of rats without a second jugular catheter were prepared as above. After 35 h, the rats were placed into individual opaque plastic boxes and a thermistor probe connected to a telethermometer (*Yellow Springs Instruments*) to record body temperature was inserted 5–6 cm into the rectum and taped to the tail (Kregel et al., 1997). The body weights and body temperatures were recorded every 15 min during acclimatization to establish baseline values. After 60 min, rats received an IP injection of NLX (1.5 mg/kg). Body temperatures and weights were recorded every 15 min for 90 min.

## Protocols to determine the effects of D-CYSee on physical dependence to morphine–Reversal of morphine dependence

### Behavioral studies

At 2 p.m. on the day of surgery, rats received a slow-release subcutaneous depot of morphine emulsion (150 mg/kg, SC) injected at the left side of the neck. After 36 h of morphine administration, the rats were anesthetized (2% isoflurane) and received a jugular vein catheter connected to a primed ALZET osmotic minipump positioned at the back of the neck to continuously infuse vehicle (20 μL/h, IV), D-cysteine, D-CYSee, D-serine or D-SERee (all at 20.8 μmol/kg/h, IV). All wounds were closed and the rats were returned to their warmed home cages. After 11.5 h, rats were placed in individual opaque plastic boxes and after 30 min, the rats received an IP injection of NLX (1.5 mg/kg) and behavioral phenomena (as detailed above) were scored for 45 min by at least three scorers.

### Plethysmography ventilatory studies

Rats were prepared as above and also received a second catheter into the jugular vein to inject NLX. After 47 h, rats were put into individual whole body plethysmography chambers and the exteriorized jugular vein catheter was connected tightly to a swivel on the chamber lid. After 60 min, rats were injected with NLX (1.5 mg/kg, IV). Ventilatory parameters and non-eupneic breathing indices were recorded with the number of apneas (>1.5 s between breaths) reported here.

## Cardiovascular studies

Rats were prepared as above except that they also received a second catheter into the jugular vein to give NLX, and a catheter into a femoral artery to record MAP and heart rate. After 47 h, the rats were placed in individual opaque plastic boxes and the free end of the exteriorized jugular vein catheter was connected to an injection line to give NLX. The arterial line was connected to tubing attached to a computer-coupled pressure transducer to record pulsatile arterial blood pressure. After a 60 min acclimatization period, the rats received an injection of NLX (1.5 mg/kg, IV) and MAP and heart rate were recorded continuously for 90 min.

## Body temperature and body weight studies

Groups of rats without a second jugular catheter were prepared as above. After 47 h, the rats were placed in individual opaque plastic boxes and a thermistor probe connected to a telethermometer (*Yellow Springs Instruments*) to record body temperature was inserted 5–6 cm into the rectum and taped to the tail. Body weights and temperatures were recorded every 15 min during acclimatization to establish baseline values. After 60 min, the rats received an intraperitoneal injection of NLX (1.5 mg/kg). Body temperatures and weights were recorded every 15 min for 90 min.

### Data analyses

All data are shown as mean ± SEM and were evaluated by one-way ANOVA followed by Bonferroni corrections for multiple comparisons between means using the error mean square terms from each ANOVA analysis (Wallenstein et al., 1980; Ludbrook, 1998; McHugh, 2011) as detailed previously (Getsy et al., 2023a; Getsy et al., 2023b). Analyses evaluated whether the number of NLX-precipitated events differed from before NLX and then evaluated differences between each treatment group. A *p* < 0.05 value denoted initial level of statistical significance modified per the number of comparisons between means. The modified *t-*statistic is t = (mean group 1–mean group 2)/[s × (1/n_1_ + 1/n_2_)^1/2^] where s^2^ = mean square within groups term from the ANOVA (the square root is used in the modified *t*-statistic formula) and n_1_ and n_2_ are rat numbers per group. Based on elementary (Bonferroni’s) inequality, a critical value for modified *t*-statistics is obtained from tables of *t*-distribution using a significance level of P/m, where m is the number of comparisons between groups (Winer, 1971). The degrees of freedom are those for the mean square for within group variation from the ANOVA table. Bonferroni values are approximated from normal curve tables by t* = z + (z + z^3^)/4n, with n being the degrees of freedom and z being the critical normal curve value for P/m. Statistics were performed with Prism software (*GraphPad Software*, Inc., La Jolla, CA).

## Results

### D-CYSee prevention of physical dependence to morphine–36 h studies

**FIGURE 1:**
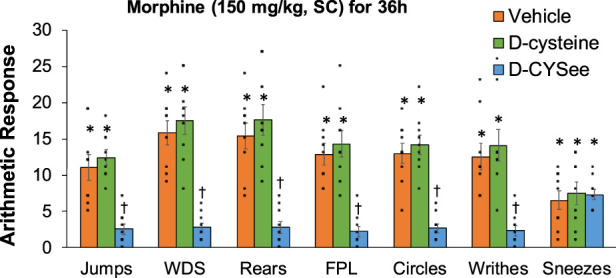
Withdrawal behaviors elicited by a bolus injection of naloxone HCl (1.5 mg/kg, IP) in rats treated for 36 h with a subcutaneous depot of morphine (150 mg/kg) along with continuous infusion of vehicle (saline, 20 μL/h, IV), D-cysteine (20.8 μmol/kg/h, IV) or D-cysteine ethyl ester (D-CYSee, 20.8 μmol/kg/h, IV). Withdrawal Signs: Jumps, all four paws off the floor; WDS, wet-dog shakes; Rears, rearing on hind legs; FPL, episodes of fore-paw licking; Circles, a 360° rotation; Writhes, fully body contortion; Sneezes, abrupt expulsion of air. The data are presented as mean ± SEM (9 rats per group). *p < 0.05, significant responses from Pre-values. ^†^ *p* < 0.05, D-CYSee *versus* vehicle or D-cysteine.

**FIGURE 2:**
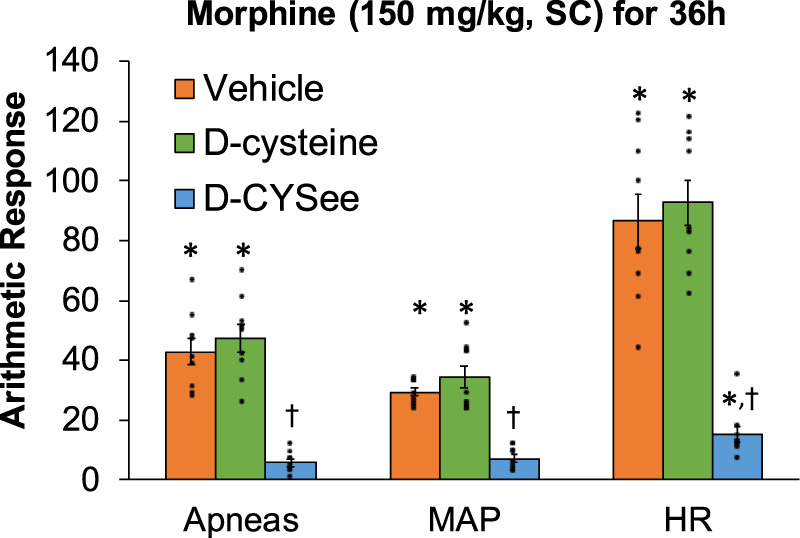
Incidence of apneas (>1.5 s), and transient increases in mean arterial blood pressure (MAP, mmHg) and heart rate (HR, beats/min) elicited by a bolus injection of naloxone HCl (1.5 mg/kg, IV) in rats treated for 36 h with a subcutaneous depot of morphine (150 mg/kg) along with continuous infusion of vehicle (saline, 20 μL/h, IV), D-cysteine (20.8 μmol/kg/h, IV) or D-cysteine ethyl ester (D-CYSee, 20.8 μmol/kg/h, IV). The data are presented as mean ± SEM (9 rats per group). *p < 0.05, significant responses from Pre-values. ^†^ *p* < 0.05, D-CYSee *versus* vehicle or D-cysteine.

Behavioral phenomena produced by NLX (1.5 mg/kg, IP) in rats receiving morphine (150 mg/kg, SC) plus infusion of vehicle (saline, 20 μL/h, IV), D-cysteine (20.8 μmol/kg/h, IV) or D-CYSee (20.8 μmol/kg/h, IV) for 36 h are shown in Figure 1. Injection of NLX in rats receiving vehicle produced jumps, wet-dog shakes (WDS), rears, fore-paw licking (FPL), circling behavior, full-body writhes and sneezes. Except for an occasional instance, these behaviors were absent prior to injection of NLX. These NLX-precipitated phenomena were similar in rats receiving D-cysteine. Withdrawal phenomena (except sneezing) were substantially reduced in rats receiving infusion of D-CYSee. NLX-precipitated withdrawal signs were reduced in rats receiving D-SERee, however, to a much lesser degree compared to D-CYSee. Additionally, the NLX-precipitated withdrawal signs were not reduced in rats receiving D-serine (Supplementary Table S3). Expression of apneas (>1.5 s between breaths), and elevations in MAP and heart rate produced by NLX in rats receiving morphine (150 mg/kg, SC), and infusion of vehicle, D-cysteine or D-CYSee are shown in Figure 2. Injection of NLX in rats that received vehicle elicited a number of apneas and elevations in MAP and heart rate (HR). These NLX-precipitated events were similar in rats receiving D-cysteine, whereas withdrawal phenomena were diminished in rats receiving D-CYSee. MAP and heart rates before and after NLX in morphine-treated rats receiving vehicle, D-cysteine or D-CYSee are shown in Supplementary Table S4. Resting MAP and heart rate values before injection of NLX were similar in the three groups of rats. Injection of NLX elevated MAP and heart rate as described above. NLX-initiated elevations in MAP and heart rate were reduced in rats receiving an infusion of D-SERee, however, to a much lesser degree compared to D-CYSee. Additionally, the NLX-initiated elevations in MAP and heart rate were not reduced in rats receiving D-serine (Supplementary Table S3).

**FIGURE 3:**
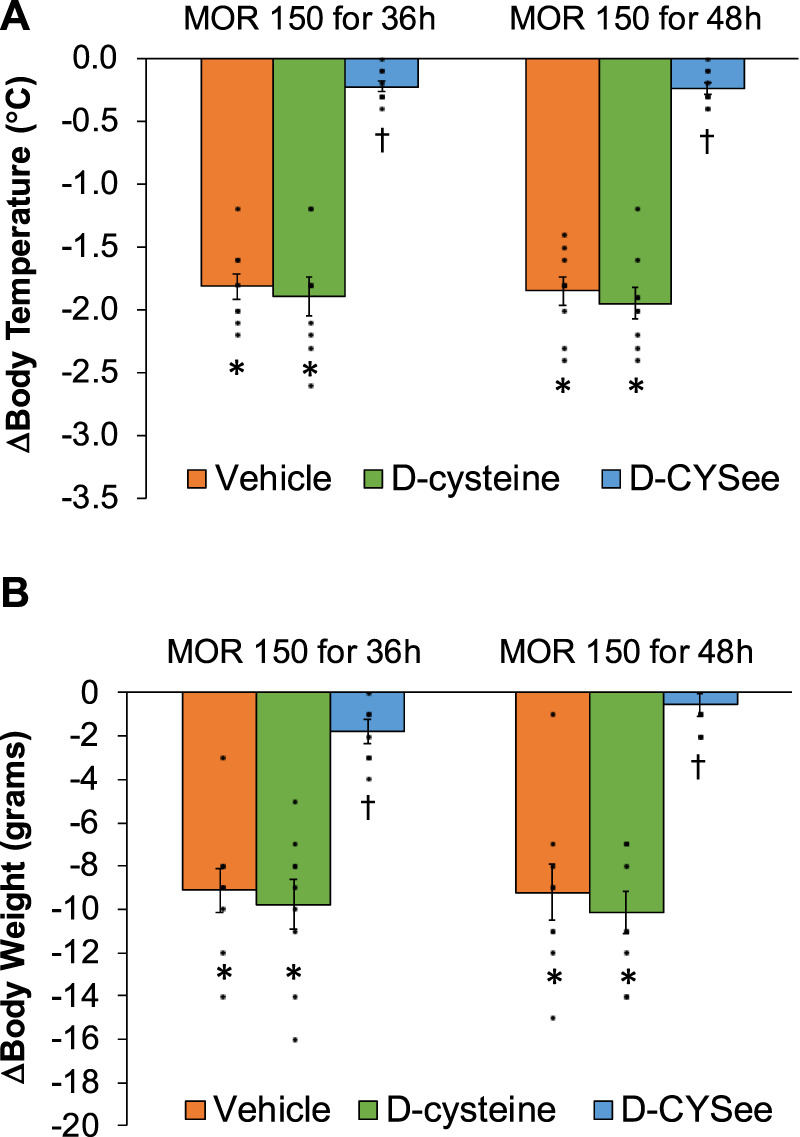
Arithmetic changes in body temperature **(A)** and body weight **(B**) elicited by a bolus injection of naloxone HCl (1.5 mg/kg, IP) in rats treated for 36 h with a subcutaneous depot of morphine (150 mg/kg) along with continuous infusion of vehicle (saline, 20 μL/h, IV), D-cysteine (20.8 μmol/kg/h, IV) or D-cysteine ethyl ester (D-CYSee, 20.8 μmol/kg/h, IV) or for 48 h with a subcutaneous depot of morphine (150 mg/kg) along with continuous infusion of vehicle (saline, 20 μL/h, IV), D-cysteine (20.8 μmol/kg/h, IV) or D-cysteine ethyl ester (D-CYSee, 20.8 μmol/kg/h, IV) that began after 36 h of morphine administration. The data are presented as mean ± SEM (9 rats per group). *p < 0.05, significant responses from Pre-values. ^†^ *p* < 0.05, D-CYSee *versus* vehicle or D-cysteine.

Decreases in body temperature and body weight elicited by NLX in rats receiving morphine (150 mg/kg, SC) and infusion of vehicle, D-cysteine or D-CYSee are shown in the left-hand panels of Figure 3. NLX decreased body temperature and body weight similarly in rats receiving infusion of vehicle or D-cysteine. These responses were smaller in rats receiving D-CYSee. Body temperature and weight before and after injection of NLX in morphine-treated rats receiving infusions of vehicle, D-cysteine or D-CYSee are shown in Supplementary Table S5. Resting values before injection of NLX were similar in the three groups. After 36 h of morphine, body temperatures were elevated by approximately 0.5°C in rats receiving vehicle or D-cysteine, whereas it was not raised in rats receiving D-CYSee. NLX elicited falls in body temperature and body weight in rats receiving vehicle or D-cysteine, and smaller responses in rats receiving D-CYSee. NLX-initiated decreases in body temperature and body weight were less in rats receiving D-SERee, however, to a much lesser degree compared to D-CYSee. Additionally, the NLX-initiated decreases in body temperature and body weight were not reduced in rats receiving D-serine (Supplementary Table S3). Body temperatures and body weights before and after injection of NLX in morphine-treated rats receiving vehicle, D-serine or D-SERee are shown in Supplementary Table S6. Resting values before NLX were similar in each group. After 36 h of morphine treatment, body temperatures were elevated by about 0.5°C in rats receiving vehicle, D-serine or D-SERee. NLX-induced decreases in body temperatures and body weights were similar in the rats receiving D-SERee compared to vehicle and D-serine.

### D-CYSee reversal of physical dependence to morphine–48 h studies

**FIGURE 4:**
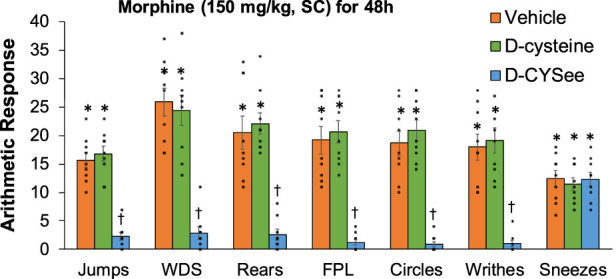
Withdrawal behaviors elicited by a bolus injection of naloxone HCl (1.5 mg/kg, IP) in rats treated for 48 h with a subcutaneous depot of morphine (150 mg/kg) along with continuous infusion of vehicle (saline, 20 μL/h, IV), D-cysteine (20.8 μmol/kg/h, IV) or D-cysteine ethyl ester (D-CYSee, 20.8 μmol/kg/h, IV) that began after 36 h of morphine administration. Withdrawal Signs: Jumps, all four paws off the floor; WDS, wet-dog shakes; Rears, rearing on hind legs; FPL, episodes of fore-paw licking; Circles, a 360° rotation; Writhes, fully body contortion; Sneezes, abrupt expulsion of air. The data are presented as mean ± SEM (9 rats per group). *p < 0.05, significant responses from Pre-values. ^†^ *p* < 0.05, D-CYSee *versus* vehicle or D-cysteine.

**FIGURE 5:**
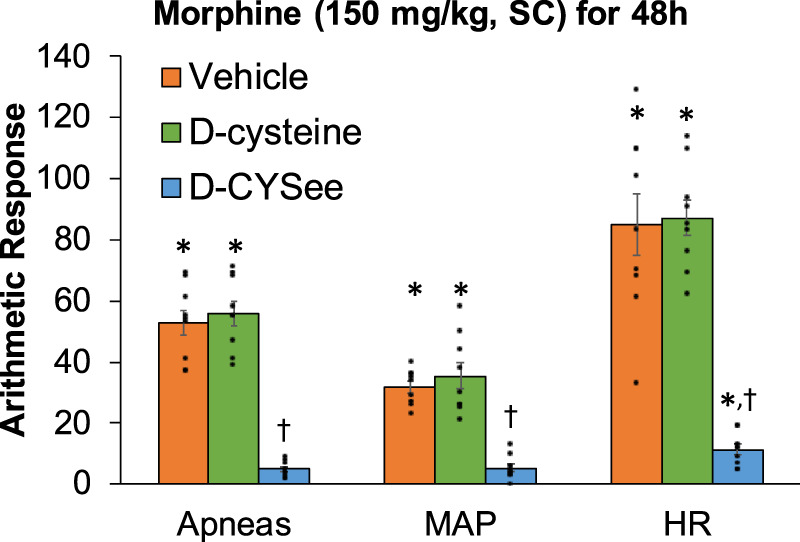
Incidence of apneas (>1.5 s), and transient repetitive increases in mean arterial blood pressure (MAP, mmHg) and heart rate (HR, beats/min) elicited by a bolus injection of naloxone HCl (1.5 mg/kg, IV) in rats treated for 48 h with a subcutaneous depot of morphine (150 mg/kg) along with continuous infusion of vehicle (saline, 20 μL/h, IV), D-cysteine (20.8 μmol/kg/h, IV) or D-cysteine ethyl ester (D-CYSee, 20.8 μmol/kg/h, IV) that began at 36 h of morphine administration. The data are presented as mean ± SEM (9 rats per group). *p < 0.05, significant responses from Pre-values. ^†^ *p* < 0.05, D-CYSee *versus* vehicle or D-cysteine.

Behavioral phenomena elicited by injection of NLX (1.5 mg/kg, IP) in rats receiving morphine (150 mg/kg, SC) for 48 h plus infusion of vehicle (saline, 20 μL/h, IV), D-cysteine (20.8 μmol/kg/h, IV) or D-CYSee (20.8 μmol/kg/h, IV) beginning at 36 h of morphine administration are shown in Figure 4. Injection of NLX to rats receiving infusion of vehicle for 12 h elicited substantial numbers of withdrawal behaviors. These phenomena were similar in rats receiving infusion of D-cysteine for 12 h, but were (except for sneezing) reduced in rats receiving D-CYSee for 12 h. NLX-precipitated behaviors in rats receiving D-serine or D-SERee were similar to those receiving vehicle (Supplementary Table S7). Incidence of apneas, and changes in MAP and heart rate produced by NLX in rats receiving morphine (150 mg/kg, SC) for 48 h and infusion of vehicle, D-cysteine or D-CYSee beginning at 36 h of morphine exposure are shown in Figure 5. NLX elicited apneas, and elevations in MAP and heart rate in rats receiving vehicle. These responses were similar in rats receiving D-cysteine, but diminished in rats receiving D-CYSee. MAP and heart rate values before and after injection of NLX in morphine-treated rats receiving vehicle, D-cysteine or D-CYSee are shown in Supplementary Table S8. Resting MAP and heart rate values before injection of NLX were similar in the three groups. NLX elicited increases in MAP and heart rate as described in Figure 5. NLX-precipitated increases in apneas, MAP and heart rate in rats receiving D-serine or D-SERee were similar to those receiving vehicle (Supplementary Table S7).

Changes in body temperature and body weight produced by NLX in rats receiving morphine (150 mg/kg, SC) for 48 h, and continuous infusion of vehicle, D-cysteine or D-CYSee for 12 h starting at 36 h of morphine exposure are shown in the right-hand panels of Figure 3. The NLX-induced decreases in body temperature and body weight were similar in rats receiving vehicle or D-cysteine. NLX-induced decreases in body temperature and body weight were smaller in rats receiving infusion of D-CYSee. Body temperature and body weight before and after injection of NLX in morphine-treated rats receiving infusions of vehicle, D-cysteine or D-CYSee are shown in Supplementary Table S9. Resting body temperature and body weight values before injection of NLX were similar in the three groups. After 48 h of morphine, body temperatures were elevated by approximately 0.6°C in rats receiving vehicle or D-cysteine. Body weights were similar in all three groups. Body temperature after 48 h or morphine was not elevated in rats receiving D-CYSee. NLX elicited falls in body temperature and body weight in vehicle- or D-cysteine-infusion groups and smaller falls in rats receiving D-CYSee. Body temperature and body weight before and after NLX in morphine-treated rats for 48 h that received continuous infusion of vehicle, D-serine or D-SERee for 12 h starting at 36 h of morphine administration are shown in Supplementary Table S10. Values before NLX were similar in each group. After 48 h of morphine, body temperatures were elevated 0.5°C in rats receiving vehicle, D-serine or D-SERee. NLX-precipitated decreases in body temperatures and body weights were similar in all three groups.

## Discussion

Co-infusion of D-CYSee reduced expression of withdrawal phenomena (behaviors, cardiorespiratory changes, body weight loss and hypothermia) initiated by injection of NLX in male Sprague Dawley rats exposed to a slow-release morphine emulsion for 36 h. Behaviors suggesting that the rats were physically dependent on morphine included wet-dog shakes, fore-paw licking, jumping, rearing, writhing, circling and sneezing. These phenomena, and the falls in body temperature and body weight were consistent with previous findings in rats using this same slow-release morphine method to induce physical dependence (Lee and Fennessy, 1970; Laska and Fennessy, 1976; Laska and Fennessy, 1977; Laska and Fennessy, 1978; Lewis et al., 1988b), and other protocols inducing morphine dependence (Hutchinson et al., 2007; Lopez-Gimenez and Milligan, 2010; Morgan and Christie, 2011; Nielsen and Kreek, 2012). The increases in MAP and heart rate elicited by NLX are novel findings in our model, but are consistent with studies showing that NLX-induced withdrawal causes hypertension and tachycardia in animals (Buccafusco, 1983; Buccafusco, 1990; Buccafusco et al., 1984; Marshall and Buccafusco, 1987; Dixon and Chang, 1988; Chang and Dixon, 1990; Delle et al., 1990; Baraban et al., 1993) and humans (Newlin et al., 1992; Purssell et al., 1995; Walsh et al., 2003; Levin et al., 2019; Balshaw et al., 2021; Isoardi et al., 2022; Lee et al., 2022) due to activation of the sympathetic nervous system. Our finding that NLX increased the incidence of apneas is new to our model, but is consistent with findings in rats (Baraban et al., 1993; Baldo et al., 2022) and humans (Schwarzer et al., 2015; Zamani et al., 2020; Wilson et al., 2023). The failure of D-cysteine to modify NLX-induced withdrawal suggests that the rapid entry of D-CYSee into cells and neurons involved in the acquisition of dependence underlies the efficacy of this cell-penetrant D-thiol ester (Laschka et al., 1976a; Laschka et al., 1976b; Laschka and Herz, 1977; Koob, 1987; Saiepour et al., 2001; Glass, 2010; Gardner, 2011; Bates et al., 2024a).

We do not know how D-CYSee modulates the central processes by which morphine induces physical dependence (Deslandes et al., 2002; Koob and Volkow, 2016; Koob, 2020; Sakloth et al., 2020). The mechanisms by which D-thiol esters act are likely to be multi-factorial (Supplementary Table S11) and involve interactions with intracellular signaling pathways involved in acquisition of physical dependence to opioids and/or the expression of NLX-precipitated withdrawal phenomena, including glutamatergic pathways using N-methyl D-aspartate (NMDA) receptors (Buccafusco et al., 1995; Herman et al., 1995; Rasmussen, 1995; Noda and Nabeshima, 2004; Glass, 2011; Fluyau et al., 2020). This study showed that D-CYSee attenuated NLX-precipitated behavioral (except sneezing), and physical (body weight loss, hypothermia) and cardiorespiratory (hypertension, tachycardia, apneas) phenomena. As such, D-CYSee may modulate intracellular processes essential to the development of physical dependence to morphine and/or those responsible for the withdrawal processes.

The second set of novel findings was that introduction of D-CYSee infusion 36 h into the morphine protocol overcame pre-existing physical dependence as assessed at 48 h. NLX-precipitated behaviors (except for sneezing), hypertension, tachycardia, apneas, hypothermia and body weight loss were diminished in rats that received D-CYSee for 12 h. The lack of effect of D-cysteine suggests that intracellular entry of D-CYSee and the thiol-associated signaling mechanisms, are essential for D-CYSee to overcome established morphine dependence. Compounds that reverse morphine dependence include, histamine receptor sub-type agonists (Wong and Roberts, 1976), melatonin (Raghavendra and Kulkarni, 1999; Raghavendra and Kulkarni, 2000), antioxidants (Singh et al., 2002; Naidu et al., 2003), a serotonin-reuptake inhibitor, fluoxetine (Singh et al., 2003), a nitric oxide synthase inhibitor (Singh et al., 2002; Naidu et al., 2003), inhibitors of Ca^2+^/calmodulin-dependent protein kinase II (Wang et al., 2003; Tang et al., 2006), a β_2_-AR antagonist, butoxamine (Liang et al., 2007), adrenomedullin receptor antagonists (Wang et al., 2011), dopamine D2 receptor antagonists (Yang et al., 2011), insulin and K_ATP_ channel modulators (Singh et al., 2015), and positive allosteric modulators of α-amino-3-hydroxy-5-methyl-4-isoxazolepropionic acid (AMPA) glutamatergic receptors (Hu et al., 2018). The finding that D-CYSee may overcome established physical dependence to morphine is of clinical relevance, and provides rationale for studies on D-CYSee and other active L,D-thiol esters to establish their ability to reverse physical dependence to morphine, heroin and fentanyl.

Potential issues related to the use of D-thiol esters as therapeutics for problems associated with long-term use of opioid analgesics in humans include, (i) if D-CYSee attenuates self-administration of opioids in OUD patients, then adding it to prescribed opioids may lower addiction/abuse potential; (ii) if D-CYSee attenuates acquisition of physical dependence to opioids, then its addition to prescribed opioids would minimize physical dependence in subjects taking opioids for weeks and months; (iii) if D-CYSee reduces development of tachyphylaxis to opioid analgesia or the switch to hyperalgesia caused by long-term opioid use, then adding D-CYSee to prescription opioids will maintain their analgesic efficacy over long periods of time, eliminating the development of tolerance, need for dose escalation, and the multiplicity of issues caused by hyperalgesia; (iv) if D-CYSee has beneficial actions seen in rats, then adding it to prescribed opioid analgesics would multiply beneficial effects of the opioids; (v) if D-CYSee prevents development of physical dependence, and if it can be given to a subject with physical dependence and can block opioid withdrawal, it could be an inpatient/outpatient drug to manage opioid withdrawal in those prescribed long-term opioid prescriptions or those physically dependent; (vi) if D-CYSee attenuates euphoria and development of psychological addiction to opioids, then it would be a much needed therapeutic for medication-assisted treatment and a valuable therapeutic for harm reduction interventions in people with OUD not willing to engage in psychosocial benefits of counseling and treatment; (vii) since some patients with a history of OUD who are currently sober need opioids for management of acute or chronic pain, then D-CYSee, if it attenuates euphoria and physical dependence, could be added to opioid analgesics given to subjects with a history of OUD, thereby eliminating risks of opioid analgesics precipitating euphoria, drug cravings and associated increased risk of relapse; (viii) if D-CYSee attenuates euphoria from drug-mediated dopamine surges in medial prefrontal cortex, nucleus accumbens or ventral tegmentum, where brain rewarding euphoria-producing dopamine surge happens from drugs of abuse (Koob and Volkow, 2016; Koob, 2020), then it will be a much needed treatment for OUD and other SUDs; (ix) if D-CYSee attenuates euphoria from chemically-mediated dopamine surges, it could be added to controlled prescription drugs resulting in an abuse-resistant or non-abusable form of prescribed opioids, benzodiazepines and psychostimulants (Koob, 1987; Saiepour et al., 2001; Deslandes et al., 2002; Glass, 2010; Gardner, 2011; Koob and Volkow, 2016; Koob, 2020; Sakloth et al., 2020).

To provide evidence that the sulfur moiety of D-CYSee and accompanying thiol biochemistry is vital to the efficacy of the D-thiol ester, we examined whether D-serine and D-SERee (oxygen for sulfur) would prevent/reverse morphine dependence. Systemic administration of D-SERee improves motor function in ataxic mice (Saigoh et al., 1998), however it does not reverse morphine-induced OIRD (Mendoza et al., 2013). In the present study, infusion of D-SERee prevented the development of dependence to morphine, whereas D-serine did not. This finding with D-serine is not consistent with evidence that co-injections of D-serine (600 mg/kg, IP) antagonize morphine (10 mg/kg, SC)-induced conditioned place preference (CPP, addiction behavioral test) in 7–9 week old rats (London et al., 1995). It is possible that our infusion paradigm (20.8 μmol/kg/h = 3.224 mg/kg/h, IV) with the highly cell-permeable D-SERee (Saigoh et al., 1998) delivers enough D-serine into key brain regions involved in dependence to morphine (Laschka et al., 1976a; Laschka et al., 1976b; Laschka and Fennessy, 1976; Laschka and Herz A. 1977; Koob, 1987; Saiepour et al., 2001; Deslandes et al., 2002; Glass, 2010; Gardner, 2011; Koob and Volkow, 2016; Koob, 2020), whereas this infusion rate of D-serine does not. The possibility that morphine induces its affects by altering the bioavailability/bioefficacy of D-serine is supported by several studies. For example, morphine increases D-serine levels in cortex, hippocampus and striatum (Yoshikawa et al., 2008), but reduces extracellular D-serine levels in nucleus accumbens, a key brain structure in opioid dependence, by blockade of Ca^2+^-dependent exocytosis of vesicular D-serine stores (Wu et al., 2017). D-serine is an endogenous NMDA receptor co-agonist (Kartvelishvily et al., 2006; Wolosker, 2007; Coyle et al., 2020). Morphine alters NMDA receptor-mediated synaptic plasticity (Wolosker, 2007; Coyle et al., 2020), reduces NMDA receptor-mediated excitatory post-synaptic currents and excitability of GABAergic neurons, and internalizes AMPA receptors (Wu et al., 2017). Several of these morphine-induced effects are reversed by D-serine (Wu et al., 2017). Although D-serine was ineffective in the present study, the ability of D-SERee to prevent acquisition of dependence to morphine adds to possible therapeutic uses of D-serine and D-SERee (Wu et al., 2017). The inability of D-SERee to overcome acquired morphine dependence suggests that D-CYSee acts *via* thiol-dependent mechanisms. Our finding with D-SERee is consistent with evidence that although co-injections of D-serine prevent morphine-induced CPP, it does not reverse established CPP (Wu et al., 2017). Evidence that D-serine is essential for opioid-withdrawal-induced long-term potentiation (opioid-induced hyperalgesia by amplification of synaptic strength at spinal C-fiber synapses after withdrawal from systemic remifentanil) suggests a complicated role for D-serine in dependence and withdrawal processes (Siggins et al., 2003; Koob and Volkow, 2010; Drdla-Schutting et al., 2019).

The development of morphine-induced dependence and addiction may involve redox-based changes in global DNA methylation and retrotransposon transcription *via* μ-OR-mediated inhibition of excitatory amino acid transporter type 3 (EAA3)-facilitated cysteine uptake into central neurons (Trivedi et al., 2014). As depicted in Figure 5 of Trivedi et al. (2014), the sequence of events proposed from published studies (Lin et al., 2001; Ikemoto et al., 2002; Mao et al., 2002; Xu et al., 2003; Xu et al., 2006; Christie, 2008; Yang et al., 2008; Wang et al., 2009; Daijo et al., 2011; Gutowicz et al., 2011; Liu et al., 2011; Maze and Nestler, 2011; Lim et al., 2012; Sun et al., 2012; Trivedi et al., 2014; Browne et al., 2020) are (i) morphine-induced reduction in uptake of L-cysteine into neurons by G protein-mediated blockade of EAA3 activity, (ii) decreases in levels of L-cysteine and L-glutathione in brain neurons, (iii) fall in S-adenosyl-methionine/S-adenosyl-homocysteine (SAM/SAH ratio, methylation index), (iv) reduced methylation of global CpG (regions of DNA in which a cytosine nucleotide is followed by a guanine nucleotide) and decreases in CpG methylation of long interspersed nuclear element-1 (LINE-1) retrotransposon regulatory regions, and (v) stimulation of transcription of previously silenced LINE-1 gene. McDonough et al. (2024) reported that overnight treatment with morphine diminished glutathione levels, induced mitochondrial damage, decreased global DNA methylation, and increased LINE-1 mRNA expression in human SH-SY5Y neurons. These adverse effects of morphine, were prevented by concurrent application of D-CYSee (100 µM) suggesting that D-CYSee prevents the appearance of redox/epigenetic signatures of opioid dependence in neural cells, which supports our *in vivo* data that D-CYSee interferes with the mechanisms responsible for opioid dependence.

### Study limitations

It is important to next test lower infusion doses of D-CYSee to find the lowest dose that effectively prevents or reverses morphine-induced physical dependence, since lower doses are less likely to have unwanted side-effects. Future studies will establish whether co-administration of D-CYSee alters the analgesic actions of long-term morphine administration, although we found that single doses of L-CYSee, and other thiol esters and related agents, prevent/reverse the actions of single injections of morphine and fentanyl on ventilatory parameters, arterial blood-gas chemistry and alveolar gas-exchange in freely-moving rats without compromising opioid analgesia or sedation (see Introduction). Synthetic opioids, such as fentanyl, are having an ever-increasing role in the on-going opioid crisis (Arendt, 2021; Deo et al., 2021), and future studies will examine whether D-CYSee is able to prevent and/or reverse physical dependence to fentanyl. A limitation is the absence of evidence about the efficacy of D-CYSee in preventing/reversing physical dependence to opioids in female rats. Opioids exert different effects on ventilatory control systems and pain in females compared to males (Dahan et al., 1998; Sarton et al., 1998; Bodnar and Kest, 2010). There are known sex differences in (1) OR-linked cell transduction processes (Bryant et al., 2006; Hosseini et al., 2011), (2) occurrence of opioid tolerance, hyperalgesia and withdrawal severity (Bodnar and Kest, 2010) and (3) expression and treatment of OUDs (Huhn et al., 2019; Davis et al., 2021; Knouse and Briand, 2021). Another limitation pertains to the distribution of D-CYSee in plasma and brain regions resulting from co-infusion of D-CYSee with vehicle or morphine. L-CYSee is readily detected in plasma, and central and peripheral structures upon systemic administration to naïve rats (Servin et al., 1988). We are performing pharmacokinetic analyses on the distribution of D-CYSee in brain regions relevant to morphine dependence by LC-MS (Altawallbeh et al., 2019). Finally, we lack meaningful information as to cellular/molecular mechanisms by which D-CYSee affects acquisition/reversal of morphine dependence. For instance, the potential mechanisms of action of D-CYSee may involve (i) direct binding to the myristoylated alanine-rich C-kinase substrate, putative D,L-cysteine binding protein (Semenza et al., 2021), (ii) interruption of μ-OR-β-arrestin-coupled cell signaling processes that do not affect the G-protein-dependent analgesic actions of morphine (Schmid et al., 2017; Grim et al., 2020), or (iii) conversion of D-CYSee to S-nitroso-D-CYSee (S-nitrosylation of the sulfur atom) *via* nitric oxide synthase-dependent mechanisms (Perissinotti et al., 2005; Hess and Stamler, 2012; Stomberski et al., 2019; Seckler et al., 2022), which may act similarly S-nitroso-L-cysteine ethyl ester (Clancy et al., 2001). Previous research has shown that redox-sensitive post-translational modifications on cysteine residues, such as S-nitrosylation and S-glutathionylation, could greatly impact structure/function of signaling proteins involved in opioid dependence (Hess and Stamler, 2012; Seckler et al., 2022).

## Conclusion

Based on our findings regarding NLX-precipitated withdrawal, the membrane-permeable D-thiol ester, D-CYSee, appeared to prevent development of physical dependence to morphine and overcome previously acquired dependence in male Sprague Dawley rats. This and our companion study with L-CYSee (Bates et al., 2023) was spurred by the work of Trivedi and co-workers who provided compelling evidence that morphine elicits physical/psychological dependence by reducing L-cysteine uptake into neurons/astrocytes by blocking the activity of the EAA3/EAAC1 transporter (Trivedi et al., 2014; Trivedi and Deth, 2015a; Trivedi et al., 2015b). Our data showing that D-CYSee reduced most of the NLX-precipitated withdrawal phenomena suggests that loss of L-cysteine entry into cells plays a key role in establishing physical dependence to morphine. Additionally, the efficacy of D-CYSee in reducing most of the NLX-precipitated withdrawal phenomena suggests that processes by which D-CYSee acts in these pathways are not stereoselective. The lone withdrawal phenomenon that was not ameliorated by D-CYSee (or L-CYSee) was sneezing, a phenomenon of opioid withdrawal in humans (Ostrea et al., 1975; Specker et al., 1998; Gaalema et al., 2012; Lofwall et al., 2013) and animals (Hendrie, 1985; Liu et al., 2007; Singh et al., 2015). The finding that D-SERee reduced sneezing precipitated by NLX in rats that received morphine for 36 h points to involvement of NMDA receptors in mechanisms responsible for sneezing during opioid withdrawal (Batsel and Lines, 1975; Undem et al., 2000; Li et al., 2021; Ramirez et al., 2022). The present findings add to knowledge of L,D-thiol esters, such as L-CYSee (Lewis et al., 2022), L-cysteine methyl ester (Getsy et al., 2022a), L-GSHee (Jenkins et al., 2021), D-CYSee (Getsy et al., 2022d,; Getsy et al., 2022e), D-cystine di(m)ethyl ester (Gaston et al., 2021) and Tempol (Baby et al., 2021a; Baby et al., 2021b), on the actions of opioids, such as fentanyl and morphine. Our findings provide rationale that L-CYSee, and other cell-permeant L-thiol esters, such as L-cysteine methyl ester, L-glutathione ethyl ester, and L-cystine diethyl ester (Getsy et al., 2022a; Supplementary Table S2), are potential drugs to prevent/overcome dependence to opioids. N-acetyl-L-cysteine (L-NAC) reduces opioid withdrawal phenomena in neonatal rats by decreasing oxidative stress in the brain (Ward et al., 2020). This suggests that the cell-penetrant L-thiol ester, L-NAC ethyl ester, a more effective antioxidant than L-NAC (Giustarini et al., 2012; Kularatne et al., 2020; Tosi et al., 2021), may be more efficacious in neonates, and an effective therapeutic in adults with OUD. Indeed, co-injections of L-NAC and L-NAC methyl ester prevent/overcome physical dependence elicited by co-injections of fentanyl in male rats (Bates et al., 2024b).

Maternal opioid use is a public health concern, and babies born to mothers dependent on opioids often show withdrawal signs severe enough to require hospitalization (Kelly et al., 2020; Centers for Disease Control and Prevention, 2023). Current therapies for neonatal opioid withdrawal syndrome (NOWS) involves opioid administration, which likely contributes to cognitive deficits and behavioral and social issues that develop later in life (Winklbaur et al., 2008; Jones et al., 2010; Reddy et al., 2017). Novel therapies and a better understanding of mechanisms that benefit immediate and long-term consequences of NOWS are needed. Phenotypes of opioid withdrawal syndrome are heritable traits subject to genetic variation (Kest et al., 2004; Philip et al., 2010). A lack of genetic variation in preclinical models is key to why findings do not translate across species (Garner, 2014; Zuberi and Lutz, 2016). Preclinical screening of potential therapeutics has provided little meaningful attention to (i) multigenic effects tested in inbred rodent strains and (ii) manipulation of specific genes causal to human pathophysiology (Mosedale, 2018). Examining therapeutic efficacies in outbred mice with 45 million segregating single nucleotide poly-morphisms (diversity similar to humans), such as the Diversity Outbred population (Saul et al., 2019; Li and Auwerx, 2020), would enhance the possibility that the therapeutic translates within species before being tested across species. In summary, D-CYSee prevented/reversed the acquisition of morphine dependence based on diminished withdrawal symptoms elicited by the OR antagonist, NLX, whereas D-cysteine was ineffective. In studies designed to define the role of thiol chemistry in the actions of D-CYSee, we found that D-SERee prevented development of dependence to morphine, but did not reverse established dependence. The inability of D-SERee to reverse morphine dependence implicates thiol/redox-dependent biochemistry in the mechanisms by which D-CYSee reverses morphine dependence. D-CYSee and analogues may be novel therapeutics that ameliorate the development/reversal of physical dependence to opioids.

## Data Availability

The raw data supporting the conclusions of this article will be made available by the authors, without undue reservation.

## Abbreviations

AMPA, α-amino-3-hydroxy-5-methyl-4-isoxazolepropionic acid; CRF, corticotropin releasing factor; EAA3, excitatory amino acid transporter; IP, intraperitoneal; IV, intravenous; L-CYSee, L-cysteine ethyl ester; L-NAC, N-acetyl-L-cysteine; Kv_1.2_ K^+^-channels, voltage-gated K^+^_1.2_ channels; L-SERee, L-serine ethyl ester; μ-OR, μ-opioid receptor; MAP, mean arterial blood pressure; NMDA, N-methyl D-aspartate; OIRD, opioid-induced respiratory depression; OUD, opioid use disorder; SAM, S-adenosyl-methionine; SAH, S-adenosyl-homocysteine; SC, subcutaneous; SUD, substance use disorder.
