# Supplementary material for: The cell-permeant antioxidant D-thiol ester D-cysteine ethyl ester overcomes physical dependence to morphine in male Sprague Dawley rats
Source: Front Pharmacol. 2024 Aug 26;15:1444574. doi: 10.3389/fphar.2024.1444574 (PMC11381264; doi:10.3389/fphar.2024.1444574)
Supplement: Supplementary file 1 [file Table1.docx]

**Supplementary File**

**The cell-permeant antioxidant D-thiol ester D-cysteine ethyl ester overcomes**

**physical dependence to morphine in male Sprague Dawley rats**

Paulina M. Getsy,^1,^* Gregory A. Coffee,^1^ James N. Bates,^2,†^ Theodore Parran,^3^ Lee Hoffer,^4^

Santhosh M. Baby,**^5^**^,‡^ Peter M. MacFarlane,^1^ Zackery T. Knauss,^6^ Derek S. Damron,^6^ Yee-Hsee Hsieh,^7^ Jason A. Bubier,^8^ Devin Mueller,^6^ and Stephen J. Lewis^1,9,10,^*

*^1^Department of Pediatrics, Case Western Reserve University, Cleveland, Ohio, USA*

*^2^Department of Anesthesiology, University of Iowa Hospitals and Clinics, Iowa City, Iowa, USA*

*^3^Center for Medical Education, Case Western Reserve University School of Medicine,*

*Cleveland, Ohio, USA*

*^4^Department of Anthropology, Case Western Reserve University, Cleveland, Ohio, USA*

*^5^Section of Biology, Galleon Pharmaceuticals, Inc, Horsham, Pennsylvania, USA*

*^6^Department of Biological Sciences, Kent State University, Kent, Ohio, USA*

*^7^Division of Pulmonary, Critical Care and Sleep Medicine, Case Western Reserve University,*

*Cleveland, Ohio, USA*

*^8^The Jackson Laboratory, Bar Harbor, Maine, USA*

*^9^Department of Pharmacology,* *Case Western Reserve University, Cleveland, Ohio, USA*

*^10^Functional Electrical Stimulation Center, Case Western Reserve University, Cleveland, Ohio, USA*

**^†^Current address:** James N. Bates, MD, PhD. Atelerix Life Sciences Inc., 300 East Main Street, Suite 202 Charlottesville, Virginia 22902, USA. Email: jnbates25@gmail.com

‡**Current address:** Santhosh M. Baby, Translational Sciences Treatment Discovery, Galvani Bioelectronics, Inc., 1250 S Collegeville Rd., Collegeville, Pennsylvania 19426. Email: santhosh.m.baby@galvani.bio

***Corresponding Author:** Paulina M. Getsy, PhD. Department of Pediatrics, Division of Pulmonology, Allergy and Immunology, School of Medicine, Case Western Reserve University, 10900 Euclid Avenue, Cleveland, OH 44106-4984. Email: pxg55@case.edu

**Supplementary Table S1**

References regarding the role of oxidative stress in the actions of drugs with substance use liability.

| **Reviews** |
| --- |
| Cunha-Oliveira T, Rego AC, Oliveira CR. Cellular and molecular mechanisms involved in the neurotoxicity of opioid and psychostimulant drugs. *Brain Res Rev*. **2008**, 58, 192-208. doi: 10.1016/j.brainresrev.2008.03.002  Salvemini D. Peroxynitrite and opiate antinociceptive tolerance: a painful reality. *Arch Biochem Biophys*. **2009**, 484, 238-44. doi: 10.1016/j.abb.2008.11.005  Salvemini D, Neumann W. Targeting peroxynitrite driven nitroxidative stress with synzymes: A novel therapeutic approach in chronic pain management. *Life Sci*. **2010**, 86, 604-614. doi: 10.1016/j.lfs.2009.06.011  Uys JD, Mulholland PJ, Townsend DM. Glutathione and redox signaling in substance abuse. *Biomed Pharmacother*. **2014**, 68, 799-807. doi: 10.1016/j.biopha.2014.06.001  Zahmatkesh M, Kadkhodaee M, Salarian A, Seifi B, Adeli S. Impact of opioids on oxidative status and related signaling pathways: An integrated view. *J. Opioid Manag*. **2017**, 13, 241-251. doi: 10.5055/jom.2017.0392.  Guleken Z, Kuruca SE, Ünübol B, Toraman S, Bilici R, Sarıbal D, Gunduz O, Depciuch J. Biochemical assay and spectroscopic analysis of oxidative/antioxidative parameters in the blood and serum of substance use disorders patients. A methodological comparison study. *Spectrochim Acta A Mol Biomol Spectrosc*. **2020**, 240, 118625. doi: 10.1016/j.saa.2020.118625.  Su LY, Liu Q, Jiao L, Yao YG. Molecular Mechanism of Neuroprotective Effect of Melatonin on Morphine Addiction and Analgesic Tolerance: an Update. Mol Neurobiol. **2021**, 58, 4628-4638. doi: 10.1007/s12035-021-02448-0.  Vorspan F, Marie-Claire C, Bellivier F, Bloch V. Biomarkers to predict staging and treatment response in opioid dependence: A narrative review. *Drug Dev Res*. **2021**, 82, 668-677. doi: 10.1002/ddr.21789  Newman M, Connery H, Boyd J. Opioids and Vitamin C: Known Interactions and Potential for Redox-Signaling Crosstalk. *Antioxidants (Basel)*. **2022**, 11, 1267. doi: 10.3390/antiox11071267  Viola TW, Orso R, Florian LF, Garcia MG, Gomes MGS, Mardini EM, Niederauer JPO, Zaparte A, Grassi-Oliveira R. Effects of substance use disorder on oxidative and antioxidative stress markers: A systematic review and meta-analysis. *Addict Biol*. **2023**, 28, e13254. doi: 10.1111/adb.13254 |
| **Opioids** |
| Payabvash S, Beheshtian A, Salmasi AH, Kiumehr S, Ghahremani MH, Tavangar SM, Sabzevari O, Dehpour AR. Chronic morphine treatment induces oxidant and apoptotic damage in the mice liver. *Life Sci*. **2006**, 79, 972-80. doi: 10.1016/j.lfs.2006.05.008  Shui HA, Ho ST, Wang JJ, Wu CC, Lin CH, Tao YX, Liaw WJ. Proteomic analysis of spinal protein expression in rats exposed to repeated intrathecal morphine injection. *Proteomics*. **2007**, 7, 796-803. doi: 10.1002/pmic.200600699  Pereska Z, Dejanova B, Bozinovska C, Petkovska L. Prooxidative/antioxidative homeostasis in **heroin** addiction and detoxification. *Bratisl Lek Listy*. **2007**, 108, 393-398.  Doyle T, Bryant L, Batinic-Haberle I, Little J, Cuzzocrea S, Masini E, Spasojevic I, Salvemini D. Supraspinal inactivation of mitochondrial superoxide dismutase is a source of peroxynitrite in the development of morphine antinociceptive tolerance. *Neuroscience*. **2009**, 164, 702-10. doi: 10.1016/j.neuroscience.2009.07.019  Ndengele MM, Cuzzocrea S, Masini E, Vinci MC, Esposito E, Muscoli C, Petrusca DN, Mollace V, Mazzon E, Li D, Petrache I, Matuschak GM, Salvemini D. Spinal ceramide modulates the development of morphine antinociceptive tolerance via peroxynitrite-mediated nitroxidative stress and neuroimmune activation. *J Pharmacol Exp* Ther. **2009**, 329, 64-75. doi: 10.1124/jpet.108.146290  Kovatsi L, Njau S, Nikolaou K, Topouridou K, Papamitsou T, Koliakos G. Evaluation of prooxidant-antioxidant balance in chronic heroin users in a single assay: an identification criterion for antioxidant supplementation. *Am J Drug Alcohol Abuse*. **2010**, 36, 228-32. doi: 10.3109/00952990.2010.495438  Bajic D, Berde CB, Commons KG. Periaqueductal gray neuroplasticity following chronic morphine varies with age: role of oxidative stress. *Neuroscience*. **2012**, 226, 165-77. doi: 10.1016/j.neuroscience.2012.09.028.  Ghazavi A, Mosayebi G, Solhi H, Rafiei M, Moazzeni SM. Serum markers of inflammation and oxidative stress in chronic opium (Taryak) smokers. *Immunol Lett*. **2013**, 153, 22-26. doi: 10.1016/j.imlet.2013.07.001  Mei B, Wang T, Wang Y, Xia Z, Irwin MG, Wong GT. High dose remifentanil increases myocardial oxidative stress and compromises remifentanil infarct-sparing effects in rats. *Eur J Pharmacol*. **2013**, 718, 484-92. doi: 10.1016/j.ejphar.2013.07.030  Motaghinejad M, Karimian SM, Motaghinejad O, Shabab B, Asadighaleni M, Fatima S. The effect of various morphine weaning regimens on the sequelae of opioid tolerance involving physical dependency, anxiety and hippocampus cell neurodegeneration in rats. *Fundam Clin Pharmacol*. **2015**, 29, 299-309. doi: 10.1111/fcp.12121  Fan R, Schrott LM, Arnold T, Snelling S, Rao M, Graham D, Cornelius A, Korneeva NL. Chronic oxycodone induces axonal degeneration in rat brain. *BMC Neurosci*. **2018**, 19, 15. doi: 10.1186/s12868-018-0417-0  Mansouri MT, Naghizadeh B, Ghorbanzadeh B, Alboghobeish S, Amirgholami N, Houshmand G, Cauli O. Venlafaxine prevents morphine antinociceptive tolerance: The role of neuroinflammation and the l-arginine-nitric oxide pathway. *Exp Neurol*. **2018**, 303, 134-141. doi: 10.1016/j.expneurol.2018.02.009  Tong J, Fitzmaurice PS, Moszczynska A, Rathitharan G, Ang LC, Meyer JH, Mizrahi R, Boileau I, Furukawa Y, McCluskey T, Sailasuta N, Kish SJ. Normal glutathione levels in autopsied brain of chronic users of heroin and of cocaine. *Drug Alcohol Depend*. **2018**, 190, 20-28. doi: 10.1016/j.drugalcdep.2018.05.021  Chen X, Zhang B, Liu T, Feng M, Zhang Y, Zhang C, Yao W, Wan L. Liproxstatin-1 Attenuates Morphine Tolerance through Inhibiting Spinal Ferroptosis-like Cell Death. *ACS Chem Neurosci*. **2019**, 10, 4824-4833. doi: 10.1021/acschemneuro.9b00539  Ajayi AF, Akhigbe RE. Codeine-induced sperm DNA damage is mediated predominantly by oxidative stress rather than apoptosis. *Redox Rep*. **2020**, 25, 33-40. doi: 10.1080/13510002.2020.1752003.  Agarwal S, Sharma H, Chen L, Dhillon NK. NADPH oxidase-mediated endothelial injury in HIV- and opioid-induced pulmonary arterial hypertension. *Am J Physiol Lung Cell Mol Physiol*. **2020**, 318, L1097-L1108. doi: 10.1152/ajplung.00480.2019  Asadi Akbarabadi E, Rajabi Vardanjani H, Molavinia S, Pashmforoosh M, Khodayar MJ. PMSF Attenuates Morphine Antinociceptive Tolerance and Dependence in Mice: Its Association with the Oxidative Stress Suppression. *Iran J Pharm Res*. **2021**, 20, 300-309. doi: 10.22037/ijpr.2020.112936.14038  Houshmand G, Pourasghar M, Shiran M, Arab Firozjae A, Goudarzi M, Manouchehr F, Shirzad S, Assadpour S, Nikbakht J, Ghorbanzadeh B. Simvastatin prevents morphine antinociceptive tolerance and withdrawal symptoms through antioxidative effect and nitric oxide pathway in mice. *Behav Brain Res*. **2021**, 402, 113104. doi: 10.1016/j.bbr.2020.113104  Pourhassanali N, Zarbakhsh S, Miladi-Gorji H. Morphine dependence and withdrawal-induced changes in mouse Sertoli cell (TM4) line: Evaluation of apoptotic, inflammatory and oxidative stress biomarkers. *Reprod Toxicol*. **2021**, 105, 175-183. doi: 10.1016/j.reprotox.2021.09.004  Rullo L, Caputi FF, Losapio LM, Morosini C, Posa L, Canistro D, Vivarelli F, Romualdi P, Candeletti S. Effects of Different Opioid Drugs on Oxidative Status and Proteasome Activity in SH-SY5Y Cells. *Molecules*. **2022**, 27, 8321. doi: 10.3390/molecules27238321  Shahidani S, Jokar Z, Alaei H, Reisi P. Effects of treadmill exercise and chronic stress on anxiety-like behavior, neuronal activity, and oxidative stress in basolateral amygdala in morphine-treated rats. *Synapse*. **2023**, 77, e22256. doi: 10.1002/syn.22256  Quintanilla ME, Morales P, Santapau D, Ávila A, Ponce C, Berrios-Cárcamo P, Olivares B, Gallardo J, Ezquer M, Herrera-Marschitz M, Israel Y, Ezquer F. Chronic Voluntary Morphine Intake Is Associated with Changes in Brain Structures Involved in Drug Dependence in a Rat Model of Polydrug Use. *Int J Mol Sci*. **2023**, 24, 17081. doi: 10.3390/ijms242317081  Makvand M, Mirtorabi SD, Campbell A, Zali A, Ahangari G. Exploring neuroadaptive cellular pathways in chronic morphine exposure: An in-vitro analysis of cabergoline and Mdivi-1 co-treatment effects on the autophagy-apoptosis axis. *J Cell Biochem*. **2024**. doi: 10.1002/jcb.30558.  Alzu'bi A, Baker WB, Al-Trad B, Zoubi MSA, AbuAlArjah MI, Abu-El-Rub E, Tahat L, Helaly AM, Ghorab DS, El-Huneidi W, Al-Zoubi RM. The impact of chronic fentanyl administration on the cerebral cortex in mice: Molecular and histological effects. *Brain Res Bull*. **2024**, 209, 110917. doi: 10.1016/j.brainresbull.2024.110917 |

**Supplementary Table S2**. Number of rats in each study group.

|  |  |  |  |  |  | **Numbers of rats in each study group** | | | | | | |
| --- | --- | --- | --- | --- | --- | --- | --- | --- | --- | --- | --- | --- |
| **Morphine treatment** |  | **Study Protocol** |  | **Drug** |  | **Behaviors** |  | **Apneas** |  | **MAP, HR** |  | **BW, BT** |
| Morphine 150 mg/kg for 36h |  | D-CYSee study |  | Vehicle |  | 9 |  | 9 |  | 9 |  | 9 |
|  |  |  |  | D-cysteine |  | 9 |  | 9 |  | 9 |  | 9 |
|  |  |  |  | D-CYSee |  | 9 |  | 9 |  | 9 |  | 9 |
|  |  | D-SERee study |  | Vehicle |  | 9 |  | 9 |  | 9 |  | 9 |
|  |  |  |  | D-serine |  | 9 |  | 9 |  | 9 |  | 9 |
|  |  |  |  | D-SERee |  | 9 |  | 9 |  | 9 |  | 9 |
| Morphine 150 mg/kg for 48h |  | D-CYSee study |  | Vehicle |  | 9 |  | 9 |  | 9 |  | 9 |
|  |  |  |  | D-cysteine |  | 9 |  | 9 |  | 9 |  | 9 |
|  |  |  |  | D-CYSee |  | 9 |  | 9 |  | 9 |  | 9 |
|  |  | D-SERee study |  | Vehicle |  | 9 |  | 9 |  | 9 |  | 9 |
|  |  |  |  | D-serine |  | 9 |  | 9 |  | 9 |  | 9 |
|  |  |  |  | D-SERee |  | 9 |  | 9 |  | 9 |  | 9 |
| **Total numbers of rats** | | | | |  | 108 |  | 108 |  | 108 |  | 108 |

The total number of rats was 432. The columns designated “Behaviors”, “Apneas”, “MAP,HR” and BW, BT” refer to the parameters measured under each protocol. D-CYSee, D-cysteine ethyl ester. D-SERee, D-serine ethyl ester. MAP, mean arterial blood pressure. HR, heart rate. BW, body weight. BT, body temperature.

**Supplementary Table S3.** Naloxone-precipitated withdrawal signs in rats treated for 36 hours with morphine and continuous infusion of vehicle, D-serine or D-serine ethyl ester.

|  |  |  | **Prevention of morphine dependence – 36h morphine** | | | | |
| --- | --- | --- | --- | --- | --- | --- | --- |
| **Study** |  | **Parameter** | **Vehicle** |  | **D-Serine** |  | **D-SERee** |
| **Study 1 - behaviors** |  | Number of rats | 9 |  | 9 |  | 9 |
|  |  | Body Weight (g) | 335 ± 2 |  | 337 ± 1 |  | 335 ± 2 |
|  |  | Jumps | 11.1 ± 1.8 |  | 12.7 ± 1.2 |  | 7.6 ± 0.9* |
|  |  | Full body wet-dog shakes | 15.9 ± 1.7 |  | 16.0 ± 1.7 |  | 9.4 ± 1.2* |
|  |  | Rearing behaviors | 15.4 ± 1.8 |  | 14.2 ± 1.4 |  | 9.1 ± 0.9* |
|  |  | Fore-paw licking | 12.9 ± 1.5 |  | 11.8 ± 1.4 |  | 7.8 ± 1.0* |
|  |  | 360^o^ Circling behavior | 13.0 ± 1.4 |  | 12.3 ± 1.6 |  | 7.3 ± 1.1* |
|  |  | Full body writhes | 12.6 ± 1.8 |  | 11.4 ± 1.6 |  | 7.0 ± 1.3* |
|  |  | Sneezes | 6.6 ± 1.3 |  | 6.3 ± 0.8 |  | 3.2 ± 0.6* |
| **Study 2 - Apneas** |  | Number of rats | 9 |  | 9 |  | 9 |
|  |  | Body Weight (g) | 336 ± 2 |  | 337 ± 2 |  | 336 ± 3 |
|  |  | Apneas | 42.8 ± 4.3 |  | 40.3 ± 3.5 |  | 26.1 ± 3.1* |
| **Study 3 - Cardiovascular** |  | Number of rats | 9 |  | 9 |  | 9 |
|  |  | Body Weight (g) | 337 ± 2 |  | 335 ± 2 |  | 336 ± 2 |
|  |  | ΔMAP (mmHg) | +29.3 ± 1.3 |  | +26.2 ± 2.7 |  | +15.3 ± 1.9* |
|  |  | ΔHeart Rate (beats/min) | +86.6 ± 9.2 |  | +80.1 ± 7.8 |  | +46.1 ± 5.3* |
| **Study 4 – Body Weight** |  | Number of rats | 9 |  | 9 |  | 9 |
| **and Body Temperature** |  | Body Weight (g) | 337 ± 2 |  | 336 ± 2 |  | 337 ± 2 |
|  |  | ΔBody Weight (g) | -9.1 ± 1.0 |  | -8.6 ± 1.2 |  | -5.7 ± 0.6* |
|  |  | ΔBody Temperature (°C) | -1.81 ± 0.10 |  | -1.63 ± 0.11 |  | -1.17 ± 0.11* |

D-SERee, D-serine ethyl ester. The data are presented as mean ± SEM. There were no between-group differences in starting body weights (*p* > 0.05, for all comparisons). **p* < 0.05, D-serine or D-SERee *versus* vehicle*.*

**Supplementary Table S4.** Changes in mean arterial blood pressure and heart rate elicited by the injection of naloxone HCl in rats treated with morphine and continuous infusion of vehicle, D-cysteine or D-CYSee for 36 hours.

| **Mean arterial blood pressure (mmHg)** | | | | |  | **Actual Values** | | | | |
| --- | --- | --- | --- | --- | --- | --- | --- | --- | --- | --- |
| **Emulsion** |  | **Infusion** |  | **Injection** |  | **Pre** |  | **+36h** |  | **Post-NLX** |
| Morphine |  | Vehicle |  | NLX |  | 112 ± 2 |  | 141 ± 3 |  | +29.3 ± 1.3* |
| Morphine |  | D-cysteine |  | NLX |  | 113 ± 1 |  | 148 ± 4 |  | +34.4 ± 3.6* |
| Morphine |  | D-CYSee |  | NLX |  | 113 ± 2 |  | 121 ± 2 |  | +7.1 ± 1.2*^,†^ |
| **Heart rate (beats/min)** | | |  |  |  | **Actual Values** | | | | |
| **Emulsion** |  | **Infusion** |  | **Injection** |  | **Pre** |  | **+36h** |  | **Post-NLX** |
| Morphine |  | Vehicle |  | NLX |  | 356 ± 7 |  | 442 ± 7 |  | +86.6 ± 9.2* |
| Morphine |  | D-cysteine |  | NLX |  | 357± 6 |  | 450 ± 7 |  | +92.8 ± 7.5* |
| Morphine |  | D-CYSee |  | NLX |  | 359 ± 8 |  | 374 ± 8 |  | +15.1 ± 2.7*^,†^ |

Responses elicited by the acute injection of naloxone HCl (1.5 mg/kg. IP) in rats treated for 36h with a subcutaneous depot of morphine (150 mg/kg) and continuous infusion of vehicle (20 μL/h, IV), D-cysteine (20.8 μmol/kg/h, IV) or D-cysteine ethyl ester (D-CYSee, 20.8 μmol/kg/h, IV). There were 9 rats in each group. The data are presented as mean ± SEM. **p* < 0.05, significant response from Pre-values. ^†^*p* < 0.05, D-cysteine or D-CYSee *versus* vehicle*.*

**Supplementary Table S5.** Changes in body temperature and body weight elicited by the injection of naloxone HCl in rats treated with morphine and continuous infusion of vehicle, D-cysteine or D-CYSee for 36 hours.

| **Body Temperature (°C)** | | | |  |  | **Actual Values** | | | | |  | **ΔChange (°C)** | | |
| --- | --- | --- | --- | --- | --- | --- | --- | --- | --- | --- | --- | --- | --- | --- |
| **Emulsion** |  | **Infusion** |  | **Injection** |  | **Pre** |  | **+36h** |  | **Post-NLX** |  | **+36h vs Pre** |  | **NLX response** |
| Morphine |  | Vehicle |  | NLX |  | 37.4 ± 0.1 |  | 37.9 ± 0.1 |  | 36.1 ± 0.1 |  | +0.52 ± 0.08* |  | -1.81 ± 0.20* |
| Morphine |  | D-cysteine |  | NLX |  | 37.4 ± 0.1 |  | 38.0 ± 0.1 |  | 36.1 ± 0.1 |  | +0.60 ± 0.09* |  | -1.89 ± 0.16* |
| Morphine |  | D-CYSee |  | NLX |  | 37.5 ± 0.1 |  | 37.6 ± 0.1 |  | 37.4 ± 0.1 |  | +0.04 ± 0.05^†^ |  | -0.22 ± 0.04*^,†^ |
| **Body Weight (grams)** | | |  |  |  | **Actual Values** | | | | |  | **ΔChange (grams)** | | |
| **Emulsion** |  | **Infusion** |  | **Injection** |  | **Pre** |  | **+36h** |  | **Post-NLX** |  | **+36h vs Pre** |  | **NLX response** |
| Morphine |  | Vehicle |  | NLX |  | 337 ± 2 |  | 337 ± 2 |  | 328 ± 1 |  | +0.4 ± 0.7 |  | -9.1 ± 1.0* |
| Morphine |  | D-cysteine |  | NLX |  | 338 ± 1 |  | 338 ± 2 |  | 328 ± 2 |  | +1.1 ± 0.7 |  | -9.8 ± 1.2* |
| Morphine |  | D-CYSee |  | NLX |  | 337 ± 1 |  | 337 ± 1 |  | 335 ± 1 |  | +1.1 ± 0.8 |  | -1.8 ± 0.5*^,†^ |

Responses elicited by the acute injection of naloxone HCl (1.5 mg/kg. IP) in rats treated for 36h with a subcutaneous depot of morphine (150 mg/kg) and continuous infusion of vehicle (20 μL/h, IV), D-cysteine (20.8 μmol/kg/h, IV) or D-cysteine ethyl ester (D-CYSee, 20.8 μmol/kg/h, IV). There were 9 rats in each group. The data are presented as mean ± SEM. **p* < 0.05, significant response from Pre-values. ^†^*p* < 0.05, D-cysteine or D-CYSee *versus* vehicle*.*

**Supplementary Table S6.** Changes in body temperature and body weight elicited by the injection of naloxone HCl in rats treated with morphine and continuous infusion of vehicle, D-serine or D-SERee for 36 hours.

| **Body Temperature (°C)** | | | |  |  | **Actual Values** | | | | |  | **ΔChange (°C)** | | |
| --- | --- | --- | --- | --- | --- | --- | --- | --- | --- | --- | --- | --- | --- | --- |
| **Emulsion** |  | **Infusion** |  | **Injection** |  | **Pre** |  | **+36h** |  | **Post-NLX** |  | **+36h vs Pre** |  | **NLX response** |
| Morphine |  | Vehicle |  | NLX |  | 37.4 ± 0.1 |  | 37.9 ± 0.1 |  | 36.1 ± 0.1 |  | +0.52 ± 0.08* |  | -1.81 ± 0.20* |
| Morphine |  | D-serine |  | NLX |  | 37.4 ± 0.2 |  | 37.9 ± 0.1 |  | 36.3 ± 0.2 |  | +0.54 ± 0.06* |  | -1.63 ± 0.11* |
| Morphine |  | D-SERee |  | NLX |  | 37.4 ± 0.1 |  | 38.0 ± 0.1 |  | 36.1 ± 0.1 |  | +0.52 ± 0.08* |  | -1.81 ± 0.10* |
| **Body Weight (grams)** | | |  |  |  | **Actual Values** | | | | |  | **ΔChange (grams)** | | |
| **Emulsion** |  | **Infusion** |  | **Injection** |  | **Pre** |  | **+36h** |  | **Post-NLX** |  | **+36h vs Pre** |  | **NLX response** |
| Morphine |  | Vehicle |  | NLX |  | 337 ± 2 |  | 337 ± 2 |  | 328 ± 1 |  | +0.4 ± 0.7 |  | -9.1 ± 1.0* |
| Morphine |  | D-serine |  | NLX |  | 336 ± 2 |  | 337 ± 2 |  | 329 ± 1 |  | +1.4 ± 0.8 |  | -8.6 ± 1.2* |
| Morphine |  | D-SERee |  | NLX |  | 337 ± 2 |  | 336 ± 2 |  | 330 ± 2 |  | +0.4 ± 0.7 |  | -9.1 ± 1.0* |

Responses elicited by the acute injection of naloxone HCl (1.5 mg/kg. IP) in rats treated for 36h with a subcutaneous depot of morphine (150 mg/kg) and continuous infusion of vehicle (20 μL/h, IV), D-serine (20.8 μmol/kg/h, IV) or D-serine ethyl ester (D-SERee,
20.8 μmol/kg/h, IV). There were 9 rats in each group. The data are presented as mean ± SEM. **p* < 0.05, significant response from Pre-values.

**Supplementary Table S7.** Naloxone-precipitated withdrawal signs in rats treated with morphine for 48 hours that received infusion of vehicle, D-serine or D-serine ethyl ester for 12 hours starting at 36 hours of morphine administration.

|  |  |  | **Reversal of morphine dependence - 48h morphine** | | | | |
| --- | --- | --- | --- | --- | --- | --- | --- |
| **Study** |  | **Parameter** | **Vehicle** |  | **D-Serine** |  | **D-SERee** |
| **Study 1 - behaviors** |  | Number of rats | 9 |  | 9 |  | 9 |
|  |  | Body Weight (g) | 336 ± 1 |  | 335 ± 1 |  | 336 ± 1 |
|  |  | Jumps | 15.7 ± 1.3 |  | 16.1 ± 1.6 |  | 15.3 ± 1.3 |
|  |  | Full body wet-dog shakes | 25.9 ± 2.5 |  | 26.0 ± 2.8 |  | 22.9 ± 2.2 |
|  |  | Rearing behaviors | 20.6 ± 2.9 |  | 19.8 ± 2.0 |  | 18.6 ± 1.3 |
|  |  | Fore-paw licking | 19.2 ± 2.4 |  | 16.3 ± 1.8 |  | 17.8 ± 1.8 |
|  |  | 360^o^ Circling behavior | 18.7 ± 2.2 |  | 17.0 ± 1.3 |  | 16.8 ± 1.3 |
|  |  | Full body writhes | 18.0 ± 2.3 |  | 17.8 ± 2.2 |  | 16.1 ± 1.3 |
|  |  | Sneezes | 12.4 ± 1.4 |  | 11.0 ± 1.8 |  | 12.1 ± 1.7 |
| **Study 2 - Apneas** |  | Number of rats | 9 |  | 9 |  | 9 |
|  |  | Body Weight (g) | 336 ± 2 |  | 336 ± 2 |  | 338 ± 2 |
|  |  | Apneas | 52.8 ± 4.1 |  | 48.8 ± 5.9 |  | 50.7 ± 5.6 |
| **Study 3 - Cardiovascular** |  | Number of rats | 9 |  | 9 |  | 9 |
|  |  | Body Weight (g) | 336 ± 2 |  | 336 ± 2 |  | 337 ± 2 |
|  |  | ΔMAP (mmHg) | +31.8 ± 1.9 |  | +27.1 ± 2.5 |  | +28.9 ± 2.1 |
|  |  | ΔHeart Rate (beats/min) | +85.0 ± 10.1 |  | +81.1 ± 6.7 |  | +77.2 ± 4.8 |
| **Study 4 – Body Weight** |  | Number of rats | 9 |  | 9 |  | 9 |
| **and Body Temperature** |  | Body Weight (g) | 337 ± 2 |  | 336 ± 2 |  | 335 ± 2 |
|  |  | ΔBody Weight (g) | -9.2 ± 1.3 |  | -8.3 ± 1.7 |  | -10.1 ± 1.2 |
|  |  | ΔBody Temperature (°C) | -1.84 ± 0.11 |  | -1.68 ± 0.18 |  | -1.87 ± 0.08 |

D-SERee, D-serine ethyl ester. The data are presented as mean ± SEM. There were no between-group differences (*p* > 0.05, for all comparisons).

**Supplementary Table S8.** Changes in mean arterial blood pressure and heart rate elicited by injection of naloxone HCl in rats treated with morphine for 48h that received infusion of vehicle, D-cysteine or D-CYSee for 12 hours starting at 36h of morphine administration.

| **Mean arterial blood pressure (mmHg)** | | | | |  | **Actual Values** | | | | |
| --- | --- | --- | --- | --- | --- | --- | --- | --- | --- | --- |
| **Emulsion** |  | **Infusion** |  | **Injection** |  | **Pre** |  | **+48h** |  | **Post-NLX** |
| Morphine |  | Vehicle |  | NLX |  | 113 ± 2 |  | 145 ± 3 |  | +31.8 ± 1.9* |
| Morphine |  | D-cysteine |  | NLX |  | 114 ± 2 |  | 149± 3 |  | +35.3 ± 4.3* |
| Morphine |  | D-CYSee |  | NLX |  | 114 ± 1 |  | 120 ± 2 |  | +5.3 ± 1.3*^,†^ |
| **Heart rate (beats/min)** | | |  |  |  | **Actual Values** | | | | |
| **Emulsion** |  | **Infusion** |  | **Injection** |  | **Pre** |  | **+48h** |  | **Post-NLX** |
| Morphine |  | Vehicle |  | NLX |  | 359 ± 7 |  | 444 ± 7 |  | +85 ± 10* |
| Morphine |  | D-cysteine |  | NLX |  | 361 ± 6 |  | 448 ± 7 |  | +87 ± 6* |
| Morphine |  | D-CYSee |  | NLX |  | 360 ± 5 |  | 372± 6 |  | +11.2 ± 1.8*^,†^ |

Responses elicited by the acute injection of naloxone HCl (1.5 mg/kg. IP) in rats treated for 48h with a subcutaneous depot of morphine (150 mg/kg) that received infusion of vehicle (20 μL/h, IV), D-cysteine (20.8 μmol/kg/h, IV) or D-cysteine ethyl ester (D-CYSee, 20.8 μmol/kg/h, IV) for 12 hours starting at 36h of morphine administration. There were 9 rats in each group. The data are presented as mean ± SEM. **p* < 0.05, significant response from Pre-values. ^†^*p* < 0.05, D-cysteine or D-CYSee *versus* vehicle*.*

**Supplementary Table S9.** Changes in body temperature and body weight elicited by the injection of naloxone HCl in rats treated with morphine for 48 hours that received continuous infusion of vehicle, D-cysteine or D-CYSee for 12 hours starting at 36 hours of morphine administration.

| **Body Temperature (°C)** | | | |  |  | **Actual Values** | | | | |  | **ΔChange (°C)** | | |
| --- | --- | --- | --- | --- | --- | --- | --- | --- | --- | --- | --- | --- | --- | --- |
| **Emulsion** |  | **Infusion** |  | **Injection** |  | **Pre** |  | **+48h** |  | **Post-NLX** |  | **+48h vs Pre** |  | **NLX response** |
| Morphine |  | Vehicle |  | NLX |  | 37.4 ± 0.1 |  | 38.1 ± 0.1 |  | 36.2 ± 0.1 |  | +0.61 ± 0.09* |  | -1.84 ± 0.11* |
| Morphine |  | D-cysteine |  | NLX |  | 37.5 ± 0.1 |  | 38.1 ± 0.1 |  | 36.2 ± 0.1 |  | +0.63± 0.11* |  | -1.94 ± 0.12* |
| Morphine |  | D-CYSee |  | NLX |  | 37.5 ± 0.1 |  | 37.5 ± 0.1 |  | 37.3 ± 0.1 |  | +0.04 ± 0.06^†^ |  | -0.23 ± 0.05*^,†^ |
| **Body Weight (grams)** | | |  |  |  | **Actual Values** | | | | |  | **ΔChange (grams)** | | |
| **Emulsion** |  | **Infusion** |  | **Injection** |  | **Pre** |  | **+48h** |  | **Post-NLX** |  | **+48h vs Pre** |  | **NLX response** |
| Morphine |  | Vehicle |  | NLX |  | 337 ± 2 |  | 338 ± 2 |  | 329 ± 2 |  | +0.4 ± 0.6 |  | -9.2 ± 1.3* |
| Morphine |  | D-cysteine |  | NLX |  | 336 ± 1 |  | 337 ± 2 |  | 326 ± 2 |  | +0.9 ± 1.0 |  | -10.1 ± 1.0* |
| Morphine |  | D-CYSee |  | NLX |  | 337 ± 1 |  | 338 ± 2 |  | 337 ± 2 |  | +0.9 ± 0.7 |  | -0.6 ± 0.5^†^ |

Responses elicited by the acute injection of naloxone HCl (1.5 mg/kg. IP) in rats treated for 48h with a subcutaneous depot of morphine (150 mg/kg) that received continuous infusion vehicle (20 μL/h, IV), D-cysteine (20.8 μmol/kg/h, IV) or D-cysteine ethyl ester (D-CYSee, 20.8 μmol/kg/h, IV) for 12 hours starting at 36 hours of morphine administration. There were 9 rats in each group. The data are presented as mean ± SEM. **p* < 0.05, significant response from Pre-values. ^†^*p* < 0.05, D-cysteine or D-CYSee *versus* vehicle*.*

**Supplementary Table S10.** Changes in body temperature and body weight elicited by the injection of naloxone HCl in rats treated with morphine for 48 hours and continuous infusion of vehicle, D-serine or D-SERee for 12 hours starting at 36 hours of morphine administration.

| **Body Temperature (°C)** | | | |  |  | **Actual Values** | | | | |  | **ΔChange (°C)** | | |
| --- | --- | --- | --- | --- | --- | --- | --- | --- | --- | --- | --- | --- | --- | --- |
| **Emulsion** |  | **Infusion** |  | **Injection** |  | **Pre** |  | **+48h** |  | **Post-NLX** |  | **+48h vs Pre** |  | **NLX response** |
| Morphine |  | Vehicle |  | NLX |  | 37.4 ± 0.1 |  | 37.9 ± 0.1 |  | 36.1 ± 0.1 |  | +0.52 ± 0.08* |  | -1.81 ± 0.20* |
| Morphine |  | D-serine |  | NLX |  | 37.4 ± 0.2 |  | 37.9 ± 0.1 |  | 36.3 ± 0.2 |  | +0.54 ± 0.06* |  | -1.63 ± 0.11* |
| Morphine |  | D-SERee |  | NLX |  | 37.4 ± 0.1 |  | 38.0 ± 0.1 |  | 36.1 ± 0.1 |  | +0.52 ± 0.08* |  | -1.81 ± 0.10* |
| **Body Weight (grams)** | | |  |  |  | **Actual Values** | | | | |  | **ΔChange (grams)** | | |
| **Emulsion** |  | **Infusion** |  | **Injection** |  | **Pre** |  | **+48h** |  | **Post-NLX** |  | **+36h vs Pre** |  | **NLX response** |
| Morphine |  | Vehicle |  | NLX |  | 337 ± 2 |  | 337 ± 2 |  | 328 ± 1 |  | +0.4 ± 0.7 |  | -9.1 ± 1.0* |
| Morphine |  | D-serine |  | NLX |  | 336 ± 2 |  | 337 ± 2 |  | 329 ± 1 |  | +1.4 ± 0.8 |  | -8.6 ± 1.2* |
| Morphine |  | D-SERee |  | NLX |  | 337 ± 2 |  | 336 ± 2 |  | 330 ± 2 |  | +0.4 ± 0.7 |  | -9.1 ± 1.0* |

Responses elicited by the acute injection of naloxone HCl (1.5 mg/kg. IP) in rats treated for 48h with a subcutaneous depot of morphine (150 mg/kg) that received continuous infusion of vehicle (20 μL/h, IV), D-serine (20.8 μmol/kg/h, IV) or D-serine ethyl ester (D-SERee,
20.8 μmol/kg/h, IV) for 12 hours starting at 36 hours of morphine administration. There were 9 rats in each group. The data are presented as mean ± SEM. **p* < 0.05, significant response from Pre-values.

**Supplementary Table S11**

| **The Mechanisms by which D-thiol esters exert their effects** |
| --- |
| The mechanisms by which D-thiol esters exert their effects are likely to be multi-factorial and may include (i) direct binding of D-CYSee to plasma membrane/intracellular proteins, such as ion-channels, receptors and enzymes that alter the activities of the proteins by mechanisms not associated with changes in redox status of the proteins, (ii) formation of thiol adducts, such as D-glucose:D-cysteine (Wróbel et al., 1997; Szwergold, 2006; Li et al., 2015), and mixed disulfides (Wilcken and Gupta, 1979; Lash and Jones, 1985; Turell et al., 2013) in the blood, (iii) modulation of redox status (e.g., reduction of disulfides to the monothiol, such as endogenous L-cystine to L-cysteine), and the activity of plasma membrane proteins, such as Kv1.2 K^+^-channels (Baronas et al., 2017), and after entry into cells, redox modulation of functional intracellular proteins (Bogeski and Niemeyer, 2014; Bogeski et al., 2011; O-Uchi Jet al., 2014; Gamper and Ooi, 2015; Gao et al., 2017; García et al., 2018), (iv) the formation of S-thiolated proteins, such as S-cysteinylated, S-cysteinylglycinylated and S-glutathionylated proteins, in plasma membranes and cells (Winkler et al., 2007; Rossi et al., 2009; Auclair et al., 2013; Baronas et al., 2017; Belcastro et al., 2017; Ghezzi and Chan P, 2017; Bonifácio et al., 2021), (v) potential conversion of D-CYSee to D-cysteine by membrane associated esterases (Butterworth et al., 1993; Nishida et al., 1996), which then enters into metabolic pathways that generate hydrogen sulfide by sequential actions of D-amino acid oxidase and 3-mercaptopyruvate sulfur-transferase in central and peripheral tissues (Kimura et al., 2014, 2017; Bełtowski et al., 2019), including the carotid bodies (Prabhakar, 2012), (vi) conversion of D-thiol esters to cysteine sulfenic, sulfinic and sulfonic acids via cysteine dioxygenase (Yamaguchi et al., 1987 ;Joseph and Maroney, 2007; Stipanuk et al., 2009, 2011), and (vii) formation of the S-nitrosothiols, S-nitroso-D-cysteine ethyl ester and S-nitroso-D-cysteine, which may behave like the endogenous S-nitrosothiol, S-nitroso-L-cysteine (Bates et al., 1991; Myers et al., 1990; Seckler et al., 2017, 2020), which has roles in intracellular signaling pathways (Lipton et al., 1993; Foster et al., 2009; Seth and Stamler, 2011; Stomberski et al., 2019; Gaston et al., 2020), including those regulating cardiovascular and ventilatory functions (Gaston et al., 2020; Davisson et al., 1996, 1997; Ohta et al., 1997; Lipton et al., 2001; Gaston et al., 2006; Lewis et al., 2006) and those blunting OIRD (Getsy et al., 2022c,f).  The above mentioned mechanisms may interact with intracellular signaling pathways specifically involved in the acquisition of physical dependence to opioids, as well as the expression of NLX-precipitated withdrawal phenomena, including pathways involving N-methyl D-aspartate (NMDA) glutamatergic receptors (Buccafusco et al., 1995; Herman et al., 1995; Rasmussen, 1995; Noda and Nabeshima, 2004 Glass, 2011; Fluyau et al., 2020), G protein-coupled muscarinic receptors (Marshall and Buccafusco, 1985; Holland et al., 1993), corticotropin releasing factor (CRF) receptor CRF1 (García-Carmona et al., 2015), tachykinin receptors (Michaud and Couture, 2003), voltage-gated Ca^2+^-channels (Tokuyama et al., 1995; Dogrul et al., 2002; Esmaeili-Mahani et al., 2008; Alboghobeish et al., 2019), adenylyl cyclase super-activation and phosphorylation of opioid receptors (Avidor-Reiss et al., 1996, 1997; Wang et al., 1999; Eckhardt et al., 2000), oxidative stress pathways (Xu et al., 2006; Mori et al., 2007; Abdel-Zaher et al., 2013; Mansouri et al., 2020; Ward et al., 2020; Houshmand et al., 2021), and nitric oxide-cGMP signaling pathways (Adams et al., 1993; Cappendijk et al., 1993; Majeed et al., 1994; Leza et al., 1995, 1996; London et al., 1995; Vaupel et al., 1995a,b; Dambisya and Lee,1996; Bhatt and Kumar, 2015; Tsakova et al., 2015; Sackner et al., 2019; Gledhill and Babey, 2021).  Because D-CYSee markedly attenuated all NLX-precipitated behavioral (except for sneezes), physical (hypothermia, body weight loss), and cardiorespiratory (hypertension, tachycardia and apneic episodes) phenomena, it is possible that D-CYSee modulates the intracellular processes that are essential to development of physical dependence to morphine in male Sprague Dawley rats. |
| **References**  Abdel-Zaher AO, Mostafa MG, Farghaly HS, Hamdy MM, Abdel-Hady RH (2013) Role of oxidative stress and inducible nitric oxide synthase in morphine-induced tolerance and dependence in mice. Effect of alpha-lipoic acid. *Behav Brain Res*. 247, 17-26. doi: 10.1016/j.bbr.2013.02.034  Adams ML, Kalicki JM, Meyer ER, Cicero TJ (1993) Inhibition of the morphine withdrawal syndrome by a nitric oxide synthase inhibitor, N^G^-nitro-L-arginine methyl ester. *Life Sci*. 52, PL245-PL249. doi: 10.1016/0024-3205(93)90472-f  Alboghobeish S, Naghizadeh B, Kheirollah A, Ghorbanzadeh B, Mansouri MT (2019) Fluoxetine increases analgesic effects of morphine, prevents development of morphine tolerance and dependence through the modulation of L-type calcium channels expression in mice. *Behav Brain Res*. 361, 86-94. doi: 10.1016/j.bbr.2018.12.020  Auclair JR, Brodkin HR, D'Aquino JA, Petsko GA, Ringe D, Agar JN (2013) Structural consequences of cysteinylation of Cu/Zn-superoxide dismutase. *Biochemistry* 52, 6145-6150. doi: 10.1021/bi400613h  Avidor-Reiss T, Nevo I, Levy R, Pfeuffer T, Vogel Z (1996) Chronic opioid treatment induces adenylyl cyclase V superactivation. Involvement of Gbetagamma. *J Biol Chem*. 1996, 271, 21309-21315. doi: 10.1074/jbc.271.35.21309  Avidor-Reiss T, Nevo I, Saya D, Bayewitch M, Vogel Z (1997) Opiate-induced adenylyl cyclase superactivation is isozyme-specific. *J Biol Chem*. 272, 5040-5047. doi: 10.1074/jbc.272.8.5040  Belcastro E, Gaucher C, Corti A, Leroy P, Lartaud I, Pompella A (2017) Regulation of protein function by S-nitrosation and S-glutathionylation: processes and targets in cardiovascular pathophysiology. *Biol Chem*. 398, 1267-1293. doi: 10.1515/hsz-2017-0150  Baronas VA, Yang RY, Kurata HT (2017) Extracellular redox sensitivity of Kv1.2 potassium channels. *Sci Rep*. 7, 9142. doi: 10.1038/s41598-017-08718-z  Bates JN, Harrison DG, Myers PR, Minor RL (1991) EDRF: nitrosylated compound or authentic nitric oxide. *Basic Res Cardiol*. 86 Suppl 2, 17-26. doi: 10.1007/978-3-642-72461-9_3  Bełtowski J (2019) Synthesis, Metabolism, and Signaling Mechanisms of Hydrogen Sulfide: An Overview. *Methods Mol Biol*. 2007, 1-8. doi: 10.1007/978-1-4939-9528-8_1  Bhatt K, Kumar A (2015) Mechanism of morphine addiction by inhibiting the soluble Guanylate Cyclase-Nitric Oxide (sGC-NO) pathway. *Math Biosci*. 266, 85-92. doi: 10.1016/j.mbs.2015.06.004  Bogeski I, Kappl R, Kummerow C, Gulaboski R, Hoth M, Niemeyer BA (2011) Redox regulation of calcium ion channels: chemical and physiological aspects. *Cell Calcium* 50, 407-423. doi: 10.1016/j.ceca.2011.07.006  Bogeski I, Niemeyer BA (2014) Redox regulation of ion channels. *Antioxid Redox Signal*. 21, 859-862. doi: 10.1089/ars.2014.6019  Bonifácio VDB, Pereira SA, Serpa J, Vicente JB (2021) Cysteine metabolic circuitries: druggable targets in cancer. *Br J Cancer* 2021, 124, 862-879. doi: 10.1038/s41416-020-01156-1  Buccafusco JJ, Terry AV, Shuster L (1995) Spinal NMDA receptor - nitric oxide mediation of the expression of morphine withdrawal symptoms in the rat. *Brain Res*. 679, 189-199. doi: 10.1016/0006-8993(95)00203-3  Butterworth M, Upshall DG, Cohen GM (1993) A novel role for carboxylesterase in the elevation of cellular cysteine by esters of cysteine. *Biochem Pharmacol*. 46, 1131-1137. doi: 10.1016/0006-2952(93)90460-e  Cappendijk SL, de Vries R, Dzoljic MR (1993) Inhibitory effect of nitric oxide (NO) synthase inhibitors on naloxone-precipitated withdrawal syndrome in morphine-dependent mice. *Neurosci Lett*. 162, 97-100. doi: 10.1016/0304-3940(93)90569-7  Dambisya YM, Lee TL (1996) Role of nitric oxide in the induction and expression of morphine tolerance and dependence in mice. *Br J Pharmacol*. 117, 914. doi: 10.1111/j.1476-5381.1996.tb15280.x.  Davisson RL, Travis MD, Bates JN, Lewis SJ (1996) Hemodynamic effects of L- and D-S-nitrosocysteine in the rat. Stereoselective S-nitrosothiol recognition sites. *Circ Res*. 79, 256-262. doi: 10.1161/01.res.79.2.256  Davisson RL, Travis MD, Bates JN, Johnson AK, Lewis SJ (1997) Stereoselective actions of S-nitrosocysteine in central nervous system of conscious rats. *Am J Physiol*. 272, H2361-H2368. doi: 10.1152/ajpheart.1997.272.5.H2361  Dogrul A, Zagli U, Tulunay FC (2002) The role of T-type calcium channels in morphine analgesia, development of antinociceptive tolerance and dependence to morphine, and morphine abstinence syndrome. *Life Sci*. 71, 725-734. doi: 10.1016/s0024-3205(02)01736-8  Eckhardt K, Nevo I, Levy R, Mikus G, Eichelbaum M, Vogel Z (2000) Morphine-related metabolites differentially activate adenylyl cyclase isozymes after acute and chronic administration. FEBS Lett. 470, 309-314. doi: 10.1016/s0014-5793(00)01329-6  Esmaeili-Mahani S, Fathi Y, Motamedi F, Hosseinpanah F, Ahmadiani A (2008) L-type calcium channel blockade attenuates morphine withdrawal: in vivo interaction between L-type calcium channels and corticosterone. *Horm Behav*. 53, 351-357. doi: 10.1016/j.yhbeh.2007.10.012  Fluyau D, Revadigar N, Pierre CG (2020) Clinical benefits and risks of N-methyl-d-aspartate receptor antagonists to treat severe opioid use disorder: A systematic review. *Drug Alcohol Depend*. 208, 107845. doi: 10.1016/j.drugalcdep.2020.107845  Foster MW, Hess DT, Stamler JS (2009) Protein S-nitrosylation in health and disease: a current perspective. *Trends Mol Med*. 15, 391-404. doi: 10.1016/j.molmed.2009.06.007  Gamper N, Ooi L (2015) Redox and nitric oxide-mediated regulation of sensory neuron ion channel function. *Antioxid Redox Signal*. 22, 486-504. doi: 10.1089/ars.2014.5884  Gao L, González-Rodríguez P, Ortega-Sáenz P, López-Barneo J (2017) Redox signaling in acute oxygen sensing. *Redox Biol*. 12, 908-915. doi: 10.1016/j.redox.2017.04.033  García-Carmona JA, Martínez-Laorden E, Milanés MV, Laorden ML (2015) Sympathetic activity induced by naloxone-precipitated morphine withdrawal is blocked in genetically engineered mice lacking functional CRF1 receptor. *Toxicol Appl Pharmacol*. 283, 42-49. doi: 10.1016/j.taap.2015.01.002  García IE, Sánchez HA, Martínez AD, Retamal MA (2018) Redox-mediated regulation of connexin proteins; focus on nitric oxide. *Biochim Biophys Acta Biomembr*. 1860, 91-95. doi: 10.1016/j.bbamem.2017.10.006  Gaston B, Singel D, Doctor A, Stamler JS (2006) S-nitrosothiol signaling in respiratory biology. *Am. J. Respir Crit Care Med*. 173, 1186-1193. doi: 10.1164/rccm.200510-1584PP  Gaston B, Smith L, Bosch J, Seckler J, Kunze D, Kiselar J, Marozkina N, Hodges CA, Wintrobe P, McGee K, Morozkina TS, Burton ST, Lewis T, Strassmaier T, Getsy P, Bates, JN, Lewis SJ (2020) Voltage-gated potassium channel proteins and stereoselective S-nitroso-l-cysteine signaling. *JCI Insight* 5, e134174. doi: 10.1172/jci.insight.134174  Getsy PM, Baby SM, Gruber RB, Gaston B, Lewis THJ, Grossfield A, Seckler JM, Hsieh YH, Bates JN, Lewis SJ (2022c) S-Nitroso-L-Cysteine Stereoselectively Blunts the Deleterious Effects of Fentanyl on Breathing While Augmenting Antinociception in Freely-Moving Rats. *Front Pharmacol*. 13, 892307. doi: 10.3389/fphar.2022.892307  Getsy PM, Young AP, Bates JN, Baby SM, Seckler JM, Grossfield A, Hsieh Y-H, Lewis THJ, Jenkins MW, Gaston B, Lewis SJ (2022f) S-nitroso-L-cysteine stereoselectively blunts the adverse effects of morphine on breathing and arterial blood gas chemistry while promoting analgesia. *Biomed Pharmacother*. 153, 113436. doi: 10.1016/j.biopha.2022.113436  Ghezzi P, Chan P (2017) Redox Proteomics Applied to the Thiol Secretome. *Antioxid Redox Signal*. 26, 299-312. doi: 10.1089/ars.2016.6732  Glass MJ (2011) Opioid dependence and NMDA receptors. *ILAR J*. 52, 342-351. doi: 10.1093/ilar.52.3.342  Gledhill LJ, Babey AM (2021) Synthesis of the Mechanisms of Opioid Tolerance: Do We Still Say NO? *Cell Mol Neurobiol*. 41, 927-948. doi: 10.1007/s10571-021-01065-8.  Herman BH, Vocci F, Bridge P (1995) The effects of NMDA receptor antagonists and nitric oxide synthase inhibitors on opioid tolerance and withdrawal. Medication development issues for opiate addiction. *Neuropsychopharmacology* 13, 269-293. doi: 10.1016/0893-133X(95)00140-9  Holland LN, Shuster LC, Buccafusco JJ (1993) Role of spinal and supraspinal muscarinic receptors in the expression of morphine withdrawal symptoms in the rat. *Neuropharmacology* 32, 1387-1395. doi: 10.1016/0028-3908(93)90035-2  Houshmand G, Pourasghar M, Shiran M, Arab Firozjae A, Goudarzi M, Manouchehr F, Shirzad S, Assadpour S, Nikbakht J, Ghorbanzadeh B (2021) Simvastatin prevents morphine antinociceptive tolerance and withdrawal symptoms through antioxidative effect and nitric oxide pathway in mice. *Behav Brain Res*. 402, 113104. doi: 10.1016/j.bbr.2020.113104  Joseph CA, Maroney MJ (2007) Cysteine dioxygenase: structure and mechanism. *Chem Commun* (*Camb*). 32, 3338-3349. doi: 10.1039/b702158e  Kimura H (2014) The physiological role of hydrogen sulfide and beyond. *Nitric Oxide* 41, 4-10. doi: 10.1016/j.niox.2014.01.002  Kimura H (2017) Hydrogen Sulfide and Polysulfide Signaling. *Antioxid. Redox Signal*. 27, 619-621. doi: 10.1089/ars.2017.7076  Lash LH, Jones DP (1985) Distribution of oxidized and reduced forms of glutathione and cysteine in rat plasma. *Arch Biochem Biophys*. 240, 583-592. doi: 10.1016/0003-9861(85)90065-7  Leza JC, Lizasoain I, San-Martín-Clark O, Lorenzo P (1995) Morphine-induced changes in cerebral and cerebellar nitric oxide synthase activity. *Eur J Pharma*col. 285, 95-98. doi: 10.1016/0014-2999(95)00474-y.  Leza JC, Lizasoain I, Cuéllar B, Moro MA, Lorenzo P (1996) Correlation between brain nitric oxide synthase activity and opiate withdrawal. *Naunyn Schmiedebergs Arch Pharmacol*. 353, 349-354. doi: 10.1007/BF00168639  Lewis SJ, Owen JR, Bates JN (2006) S-nitrosocysteine elicits hemodynamic responses similar to those of the Bezold-Jarisch reflex via activation of stereoselective recognition sites. *Eur J Pharmaco*l. 531, 254-258. doi: 10.1016/j.ejphar.2005.11.027  Li Y, Su L, Li F, Wang C, Yuan D, Chen J, Tan L, Jin Z, Ma W (2015) Acute and sub-chronic toxicity of glucose-cysteine Maillard reaction products in Sprague-Dawley rats. *Food Chem Toxicol*. 80, 271-276. doi: 10.1016/j.fct.2015.03.021  Lipton SA, Choi YB, Pan ZH, Lei SZ, Chen HS, Sucher NJ, Loscalzo J, Singel DJ, Stamler JS (1993) A redox-based mechanism for the neuroprotective and neurodestructive effects of nitric oxide and related nitroso-compounds. *Nature* 364, 626-632. doi: 10.1038/364626a0  Lipton AJ, Johnson MA, Macdonald T, Lieberman MW, Gozal D, Gaston B (2001) S-nitrosothiols signal the ventilatory response to hypoxia. *Nature* 413, 171-174. doi: 10.1038/35093117  London ED, Kimes AS, Vaupel DB (1995) Inhibitors of nitric oxide synthase and the opioid withdrawal syndrome. *NIDA Res Monogr*. 147, 170-181.  Majeed NH, Przewłocka B, Machelska H, Przewłocki R (1994) Inhibition of nitric oxide synthase attenuates the development of morphine tolerance and dependence in mice. *Neuropharmacology* 33, 189-192. doi: 10.1016/0028-3908(94)90006-x  Mansouri MT, Naghizadeh B, Ghorbanzadeh B, Amirgholami N, Houshmand G, Alboghobeish S (2020) Venlafaxine inhibits naloxone-precipitated morphine withdrawal symptoms: Role of inflammatory cytokines and nitric oxide. *Metab Brain* Dis. 35, 305-313. doi: 10.1007/s11011-019-00491-4  Marshall DC, Buccafusco JJ (1985) Supraspinal and spinal mediation of naloxone-induced morphine withdrawal in rats. *Brain Res*. 329, 131-142. doi: 10.1016/0006-8993(85)90518-9  Michaud N, Couture R (2003) Cardiovascular and behavioural effects induced by naloxone-precipitated morphine withdrawal in rat: characterization with tachykinin antagonists. *Neuropeptides* 37, 345-354. doi: 10.1016/j.npep.2003.09.003.  Mori T, Ito S, Matsubayashi K, Sawaguchi T (2007) Comparison of nitric oxide synthase inhibitors, phospholipase A2 inhibitor and free radical scavengers as attenuators of opioid withdrawal syndrome. *Behav Pharmacol*. 18, 725-729. doi: 10.1097/FBP.0b013e3282f18da6.  Myers PR, Minor RL Jr, Guerra R Jr, Bates JN, Harrison DG (1990) Vasorelaxant properties of the endothelium-derived relaxing factor more closely resemble S-nitrosocysteine than nitric oxide. *Nature* 345, 161-163. doi: 10.1038/345161a0  Nishida K, Ohta Y, Ito M, Nagamura Y, Kitahara S, Fujii K, Ishiguro I (1996) Conversion of gamma-glutamylcysteinylethyl ester to glutathione in rat hepatocytes. *Biochim Biophys Acta* 1313, 47-53. doi: 10.1016/0167-4889(96)00054-7  Noda Y, Nabeshima T (2004) Opiate physical dependence and N-methyl-D-aspartate receptors. *Eur J Pharmacol*. 500, 121-128. doi: 10.1016/j.ejphar.2004.07.017  Ohta H, Bates JN, Lewis SJ, Talman WT (1997) Actions of S-nitrosocysteine in the nucleus tractus solitarii are unrelated to release of nitric oxide. *Brain Res*. 746, 98-104. doi: 10.1016/s0006-8993(96)01188-2  O-Uchi J, Ryu SY, Jhun BS, Hurst S Sheu SS (2014) Mitochondrial ion channels/transporters as sensors and regulators of cellular redox signaling. *Antioxid Redox Signal*. 21, 987-1006. doi: 10.1089/ars.2013.5681  Rasmussen K (1995) The role of the locus coeruleus and N-methyl-D-aspartic acid (NMDA) and AMPA receptors in opiate withdrawal. *Neuropsychopharmacology* 13, 295-300. doi: 10.1016/0893-133X(95)00082-O  Rossi R, Giustarini D, Milzani A, Dalle-Donne I (2009) Cysteinylation and homocysteinylation of plasma protein thiols during ageing of healthy human beings. *J Cell Mol Med*. 13, 3131-3140. doi: 10.1111/j.1582-4934.2008.00417.x  Sackner MA, Lopez JR, Banderas V, Adams JA (2019) Holistic approach to opioid use disorder: Think nitric oxide! *J Opioid Manag*. 15, 521-555. doi: 10.5055/jom.2019.0543  Seckler JM, Meyer NM, Burton ST, Bates JN, Gaston B, Lewis SJ (2017) Detection of trace concentrations of S-nitrosothiols by means of a capacitive sensor. *PLoS One* 12, grime0187149. doi: 10.1371/journal.pone.0187149  Seckler JM, Shen J, Lewis THJ, Abdulameer MA, Zaman K, Palmer LA, Bates JN, Jenkins MW, Lewis SJ (2020) NADPH diaphorase detects S-nitrosylated proteins in aldehyde-treated biological tissues. *Sci Rep*. 10, 21088. doi: 10.1038/s41598-020-78107-6  Seth D, Stamler JS (2011) The SNO-proteome: causation and classifications. *Curr Opin Chem Biol*. 15, 129-136. doi: 10.1016/j.cbpa.2010.10.012  Stipanuk MH, Ueki I, Dominy JE Jr, Simmons CR, Hirschberger LL (2009) Cysteine dioxygenase: a robust system for regulation of cellular cysteine levels. *Amino Acids* 37, 55-63. doi: 10.1007/s00726-008-0202-y  Stipanuk MH, Simmons CR, Karplus PA, Dominy, JE Jr (2011) Thiol dioxygenases: unique families of cupin proteins. *Amino Acids* 41, 91-102. doi: 10.1007/s00726-010-0518-2  Stomberski CT, Hess DT, Stamler JS (2019) Protein S-Nitrosylation: Determinants of Specificity and Enzymatic Regulation of S-Nitrosothiol-Based Signaling. *Antioxid Redox Signal*. 30, 1331-1351. doi: 10.1089/ars.2017.7403  Szwergold BS (2006) Alpha-thiolamines such as cysteine and cysteamine act as effective transglycating agents due to formation of irreversible thiazolidine derivatives. *Med Hypotheses* 66, 698-707, 2006.  Tokuyama S, Feng Y, Wakabayashi H, Ho IK (1995) Ca^2+^ channel blocker, diltiazem, prevents physical dependence and the enhancement of protein kinase C activity by opioid infusion in rats. *Eur J Pharmacol*. 279, 93-98. doi: 10.1016/0014-2999(95)00140-g  Tsakova A, Surcheva S, Simeonova K, Altankova I, Marinova T, Usunoff K, Vlaskovska M (2015) Nitroxidergic modulation of behavioural, cardiovascular and immune responses, and brain NADPH diaphorase activity upon morphine tolerance/dependence in rats. *Biotechnol Biotechnol Equip*. 2015, 29, 92-100. doi: 10.1080/13102818.2014.990924  Turell L, Radi R, Alvarez B (2013) The thiol pool in human plasma: the central contribution of albumin to redox processes. *Free Radic Biol Med*. 65, 244-253. doi: 10.1016/j.freeradbiomed.2013.05.050  Vaupel DB, Kimes AS, London ED (1995a) Nitric oxide synthase inhibitors. Preclinical studies of potential use for treatment of opioid withdrawal. *Neuropsychopharmacology* 13, 315-322. doi: 10.1016/0893-133X(95)00138-4  Vaupel DB, Kimes AS, London ED (1995b) Comparison of 7-nitroindazole with other nitric oxide synthase inhibitors as attenuators of opioid withdrawal. *Psychopharmacology* (*Berl*) 118, 361-368. doi: 10.1007/BF02245935  Wang Z, Bilsky EJ, Wang D, Porreca F, Sadée W (1999) 3-Isobutyl-1-methylxanthine inhibits basal mu-opioid receptor phosphorylation and reverses acute morphine tolerance and dependence in mice. *Eur J Pharmacol*. 371, 1-9. doi: 10.1016/s0014-2999(99)00131-4  Ward P, Moss HG, Brown TR, Kalivas P, Jenkins DD (2020) N-acetylcysteine mitigates acute opioid withdrawal behaviors and CNS oxidative stress in neonatal rats. *Pediatr Res*. 88, 77-84. doi: 10.1038/s41390-019-0728-6  Wilcken DE, Gupta VJ (1979) Cysteine-homocysteine mixed disulphide: differing plasma concentrations in normal men and women. *Clin Sci* (*Lond*). 57, 211-215. doi: 10.1042/cs0570211  Winkler A, Kutchan TM, Macheroux P (2007) 6-S-cysteinylation of bi-covalently attached FAD in berberine bridge enzyme tunes the redox potential for optimal activity. *J Biol Chem*. 282, 24437-24443. doi: 10.1074/jbc.M703642200  Wróbel M, Ubuka T, Yao WB, Abe T (1997) Effect of glucose-cysteine adduct on cysteine desulfuration in guinea pig tissues. *Physiol Chem Phys Med NMR*. 29, 11-14.  Xu B, Wang Z, Li G, Li B, Lin H, Zheng R, Zheng Q (2006) Heroin-administered mice involved in oxidative stress and exogenous antioxidant-alleviated withdrawal syndrome. *Basic Clin Pharmacol Toxicol*. 99, 153-161. doi: 10.1111/j.1742-7843.2006.pto_461.x  Yamaguchi K, Hosokawa Y (1987) Cysteine dioxygenase. *Methods Enzymol*. 1987, 143, 395-403. doi: 10.1016/0076-6879(87)43069-3 |
